# Integrated taxonomy of two new species of the millipede genus *Plusioglyphiulus* Silvestri, 1923 from Cambodia (Diplopoda, Spirostreptida)

**DOI:** 10.3897/zookeys.1289.204580

**Published:** 2026-08-14

**Authors:** Natdanai Likhitrakarn, Sergei I. Golovatch, Ruttapon Srisonchai, Sothearen Thi, Sophea Chhin, Vanny Lou, Pablo Sinovas, Parin Jirapatrasilp, Chirasak Sutcharit, Somsak Panha, Teerapong Seesamut

**Affiliations:** 1 Division of Plant Protection, Faculty of Agricultural Production, Maejo University, Chiang Mai 50290, Thailand Department of Biology, Faculty of Science, Rangsit University Pathumthani Thailand; 2 A. N. Severtsov Institute of Ecology and Evolution, Russian Academy of Sciences, Leninsky pr. 33, Moscow 119071, Russia Animal Systematics Research Unit, Department of Biology, Faculty of Science, Chulalongkorn University Bangkok Thailand; 3 Department of Biology, Faculty of Science, Khon Kaen University, Khon Kaen 40002, Thailand Division of Plant Protection, Faculty of Agricultural Production, Maejo University Chiang Mai Thailand; 4 Fauna & Flora Cambodia Programme, 19 Street 360, Phnom Penh, Cambodia Department of Biology, Faculty of Science, Khon Kaen University Khon Kaen Thailand; 5 Department of Biodiversity, General Directorate of Policy and Strategy, Ministry of Environment, Phnom Penh, Cambodia Academy of Science, The Royal Society of Thailand Bangkok Thailand; 6 Animal Systematics Research Unit, Department of Biology, Faculty of Science, Chulalongkorn University, Bangkok 10330, Thailand A. N. Severtsov Institute of Ecology and Evolution, Russian Academy of Sciences Moscow Russia; 7 Academy of Science, The Royal Society of Thailand, Bangkok 10300, Thailand Fauna & Flora Cambodia Programme Phnom Penh Cambodia; 8 Department of Biology, Faculty of Science, Rangsit University, Pathumthani 12000, Thailand Department of Biodiversity, General Directorate of Policy and Strategy, Ministry of Environment Phnom Penh Cambodia

**Keywords:** Cambalopsidae, COI, DNA barcoding, identification key, Indochina, limestone karst

## Abstract

Two new sympatric cavernicolous species of the millipede genus *Plusioglyphiulus* Silvestri, 1923, are described from Battambang Province, northwestern Cambodia: *Plusioglyphiulus parviserratus* Likhitrakarn & Seesamut, **sp. nov**. and *Plusioglyphiulus battambangensis* Likhitrakarn & Seesamut, **sp. nov**. Both new taxa are diagnosed based on an integrated taxonomic approach combining morphological features and mitochondrial cytochrome *c* oxidase subunit I (COI) gene sequences from a previous study. *Plusioglyphiulus parviserratus***sp. nov**. is distinguished from all congeners by having the male leg 1 telopodite distinctly 2-segmented with a rudimentary, knob-like second telopoditomere, the anterior gonopod coxosternal processes long, slender, and regularly curved anteriorly, resembling a bird’s head, and a unique micro-serrate upper margin of the broad flagellum process. In contrast, *P. battambangensis***sp. nov**. is characterized by a distinct carinotaxic formula of the collum, a significantly smaller second telopoditomere on the male leg 1, a slender, elongated, paramedian coxal process of the posterior gonopod, and a narrow, smooth-margined flagellum process. The two new species are separated from each other by an interspecific COI p-distance of 10.3% and show divergences of 9.6–9.9% from their closest known relatives, all lying well within the stable range observed for species boundaries in the Cambalopsidae and therefore strongly supporting their independent specific status.

Beyond traditional descriptions, this study evaluates the resolution efficiency and practical limitations of multiple imaging modalities, including the standard transmission light photography, traditional line drawings, scanning electron microscopy, and micro-computed tomography scanning. While 3DµCT reconstructions provide powerful, non-destructive visualizations of external somatic architectures, we demonstrate that current in-situ tomographic data remain insufficient for resolving the highly condensed, tightly appressed gonopodial microscopic characters, thereby emphasizing the continued necessity of physical dissection and SEM in cambalopsid taxonomy.

These findings increase the number of *Plusioglyphiulus* species currently known to occur in Cambodia to six, highlighting the significant, yet severely understudied, cavernicolous diplopod diversity in the country’s extensive karst systems. An updated identification key to all six Cambodian *Plusioglyphiulus* species is provided. These discoveries underscore the critical need for continued taxonomic exploration and the implementation of conservation strategies for the imperiled limestone karst ecosystems in the region.

## Introduction

The millipede genus *Plusioglyphiulus* Silvestri, 1923, restricted to Southeast Asia, is one of the most conspicuous, species-rich, widespread, and frequently abundant groups dominating the cavernicolous fauna of the region (Golovatch 2015). To date, the genus comprises 30 described species ranging from southern Myanmar, Thailand, and Laos in the west to Borneo in the east and southeast (Golovatch et al. 2009, 2011; Likhitrakarn et al. 2018, 2020a). The highest diversity has been recorded from Thailand, which alone harbors 14 species (Golovatch et al. 2011; Likhitrakarn et al. 2023), followed by Malaysia and Indonesia (seven species combined from the Malay Peninsula and Borneo) (Golovatch et al. 2009), Cambodia (four species) (Likhitrakarn et al. 2020a), and Laos (three species) (Golovatch et al. 2009). In contrast, Myanmar and Vietnam are currently known to support only a single species each (Golovatch et al. 2009; Likhitrakarn et al. 2018; Nguyen et al. 2025). From an evolutionary perspective, the Cambalopsidae represents a remarkably ancient lineage. This is evidenced by the recently described *Laeviglyphiulus patrickmuelleri* Wesener & Rühr, 2025, an amber-preserved fossil from the Late Cretaceous of Myanmar (ca 99 Mya) which exhibits closer morphological affinities to the extant genus *Hypocambala* Silvestri, 1897, than to *Plusioglyphiulus* (Wesener and Moritz 2017; Wesener and Rühr 2025).

*Plusioglyphiulus* species are strongly related to karst landscapes, which are long recognized as biodiversity hotspots that are threatened in Southeast Asia (Clements et al. 2006). Most congeners show a significant degree of micro-endemism due to their limited dispersal capabilities and specific habitat requirements. Species tend to generally be confined to a single cave or a solitary limestone outcrop (Golovatch et al. 2009, 2011; Likhitrakarn et al. 2020a). Even though narrow endemism is common, there are some important exceptions where species occupy more than one cave next to each other. For example, *P. erawan* Golovatch, Geoffroy, Mauriès & VandenSpiegel, 2011 in northern Thailand (Golovatch et al. 2011; Likhitrakarn et al. 2023) and *P. digitiformis* Likhitrakarn, Golovatch, Srisonchai, Brehier, Lin, Sutcharit & Panha, 2018 in southern Myanmar (Likhitrakarn et al. 2018). In most circumstances, a single cave usually supports only one cambalopsid species. However, there is a rare case of syntopy in Borneo, where *P. bedosae* Golovatch, Geoffroy, Mauriès & VandenSpiegel, 2009 and *P. pallidior* Golovatch, Geoffroy, Mauriès & VandenSpiegel, 2009 coexist in one cave (Golovatch et al. 2009). The significant size difference between these two syntopic congeners strongly suggests niche segregation to minimize resource competition (Golovatch et al. 2009). Notably, despite frequently forming massive populations within caves, nearly all *Plusioglyphiulus* species lack troglomorphic features and are thus regarded as troglophiles (Golovatch et al. 2011; Likhitrakarn et al. 2018), with bat guano serving as the primary nutrient source in these subterranean ecosystems (Golovatch 2015).

Even though large karst limestone areas blanket Cambodia’s western and southern parts, they are not too thoroughly surveyed yet for their cave fauna (Clements et al. 2006; Likhitrakarn et al. 2015). As a result, the country remains dramatically understudied for its diplopod diversity, especially when compared to the neighboring Thailand which currently supports as many as 14 recorded *Plusioglyphiulus* species, mostly cavernicolous (Likhitrakarn et al. 2023). Until recently, only two species of this genus had hitherto been documented from Cambodia: *P. dubius* (Attems, 1938) and *P. boutini* Mauriès, 1970, both being presumably endemic to the country. It was not until 2020 that a field survey yielded another two new cavernicolous congeners: *P. biserratus* Likhitrakarn, Golovatch, Thach, Chhuoy, Ngor, Srisonchai, Sutcharit & Panha, 2020 and *P. khmer* Likhitrakarn, Golovatch, Thach, Chhuoy, Ngor, Srisonchai, Sutcharit & Panha, 2020, from the southern provinces of Kampot and Kep, respectively (Likhitrakarn et al. 2020a). Consequently, the present list of Cambodian *Plusioglyphiulus* comprises only four species. Such a paucity of records undoubtedly reflects the severe lack of collecting efforts and taxonomic explorations in Cambodia’s extensive karst systems, rather than the true biogeographical patterns of this genus.

The main objective of the present paper is to describe and illustrate further two new cavernicolous species of *Plusioglyphiulus* from Cambodia, both recently discovered from karst cave ecosystems in Battambang province, respectively, and recognized as distinct evolutionary lineages in the previous study (Seesamut et al. 2026: fig. 1). The present study also explores a multi-faceted imaging approach to document the delicate somatic and gonopodal structures of these millipedes. By integrated application and comparison using the standard transmission light photography, detailed line drawings, scanning electron microscopy (SEM), and micro-computed tomography (µCT) scanning, we further aim to evaluate the resolution efficiency and cost-effectiveness of each technique, thereby suggesting an optimal visualization pipeline for future taxonomic work on this millipede group. Furthermore, to enhance the value of this contribution for future research, we provide an updated identification key to all six species of *Plusioglyphiulus* currently known to occur in Cambodia. These discoveries not only fill in the existing biogeographical gaps for this genus but also strongly reiterate the urgent need to evaluate and drive conservation strategies for the imperiled karst ecosystems in the region.

## Material and methods

### Sample collection

Specimens were obtained by hand-collecting from karst landscapes in Battambang Province, Cambodia. Geographical coordinates and elevations were recorded *in situ* using a Garmin GPSMAP 60CSx receiver (WGS84 datum), with all locality data subsequently verified via Google Earth Pro v. 7.3.6. Live animals were photographed in their natural habitats using a Canon EOS 90D digital camera equipped with a Canon EF-S 60 mm f/2.8 Macro USM lens.

Fieldwork and sampling were conducted under the approval of the Animal Care and Use Committee of the Chulalongkorn University Animal Care and Use Committee (Protocol Review No. 1723018). Specimens were euthanized following the two-step method as per the AVMA Guidelines for the Euthanasia of Animals (AVMA 2020). For morphological studies, material was preserved in 70% ethanol, while specimens intended for molecular analysis were stored in 95% ethanol. In the latter case, the preservative was replaced with fresh 95% ethanol after 24 h to prevent defensive secretions from compromising DNA quality. All type specimens are deposited in the Museum of Zoology, Chulalongkorn University (**CUMZ**), Bangkok, Thailand.

### Morphological study and scanning electron microscopy

Specimens were examined, measured, and illustrated using a Nikon SMZ 745T trinocular stereo microscope equipped with a Canon EOS 5DS R digital camera. Digital stereomicroscopic photography was executed utilizing Leica M205 FCA and Leica M205 FA fluorescence stereo microscopes, with image acquisition and focus-stacked data processing mediated via the LAS X microscope software suite. Traditional line drawings were subsequently prepared based on these focus-stacked digital renderings using a Leica DM500 microscope coupled with a Leica DMC2900 digital USB 3.0 microscope camera, the latter equipped with a 3.1 Megapixel CMOS sensor. To resolve critical, otherwise visually elusive micro-characters, observations and photography were further enhanced by means of phase-contrast microscopy utilizing a Leica HC PL Fluotar 63×/1.30 Oil Immersion Objective (contrast plan fluorite objective series). All digital images were finally processed and arranged into plates deploying Adobe Photoshop CS6. The initial line drawings were subsequently finalized based on these stacked photographs and further refined through rigorous, direct microscopic observations.

Descriptive terminology, including the carinotaxic formulae, follows the standards established by Golovatch et al. (2007a, 2007b, 2009, 2011). Body ring counts are in accordance with the methods used by Enghoff et al. (1993) and Golovatch et al. (2007a). In the catalogue sections, **D** stands for the original description and subsequent descriptive notes; **K** for the appearance in a key; **L** for the appearance in a species list; **R** for new subsequent records from Cambodia; **M** for a mere mention; and **MI** for molecular information. The abbreviations used for specific gonopodal structures are as follows:

**ap** = anterior coxal process;

**cxp 1** = anterior coxosternal processes;

**cxp 2** = posterior coxosternal processes;

**f** = flagellum process;

**pp** = paramedian coxal process;

**te** = telopodite.

For scanning electron microscopy (SEM), gonopods were mounted on aluminum stubs using conductive carbon tape and coated with a 5-nm platinum layer using a CCU-010 high vacuum sputter and a carbon coater (Safematic). Images were captured with a TESCAN VEGA3 scanning electron microscope operated at an acceleration voltage of 5 keV and a working distance of 25 mm using a secondary electron (SE) detector. Following SEM examination, gonopods were carefully removed from the stubs, rinsed in acetone to remove tape residue, and returned to 75% ethanol for long-term deposition in the museum collection.

### Micro-computed tomography and 3D virtual reconstruction

Micro-CT imaging was performed using a SkyScan 1273 scanner (Bruker, Kontich, Belgium) operated at a source voltage of 40 kV and a source current of 50 μA without an X-ray filter. Images were acquired with a voxel size of 4.0 μm, a camera binning of 1×1, and a rotation step of 0.3° for a full 360° scan. The projection images were reconstructed using NRecon software (v. 2.2.0.6, Bruker). Two-dimensional (2D) images were processed and visualized using DataViewer software (v. 1.5.6.2, Bruker) and three-dimensional (3D) renderings were generated using CTvox software (v. 3.3.1, Bruker).

Volumetric 3D reconstructions of the computed tomography scans were compiled using the software XMReconstructor (v. 10.7.2936), with the resulting default parameters exported and saved in the standard 16-bit USHORT DICOM file format. Subsequent rendering and fine processing of the raw tomographic data were executed via AIVIA (v. 15). For detailed morphological examinations, 3D surface configurations were rendered using the volume rendering or “volren” processing options. The final virtual models were generated by strictly minimizing the color space thresholds, thereby guaranteeing that the superficial ectal cuticular structures of the specimens remained perfectly visible at the highest attainable resolution. The virtual reconstructions were dynamically rotated and manipulated along all anatomical axes to permit an exhaustive, non-destructive examination of the scanned material. Subsequently, the snapshot function of the software was used to capture high-resolution figures of the shaded surface volumes, which provided clear figures at a standard resolution of approximately 1281 × 732 pixels.

### Molecular framework and genetic distance analyses

**Figure 1.**
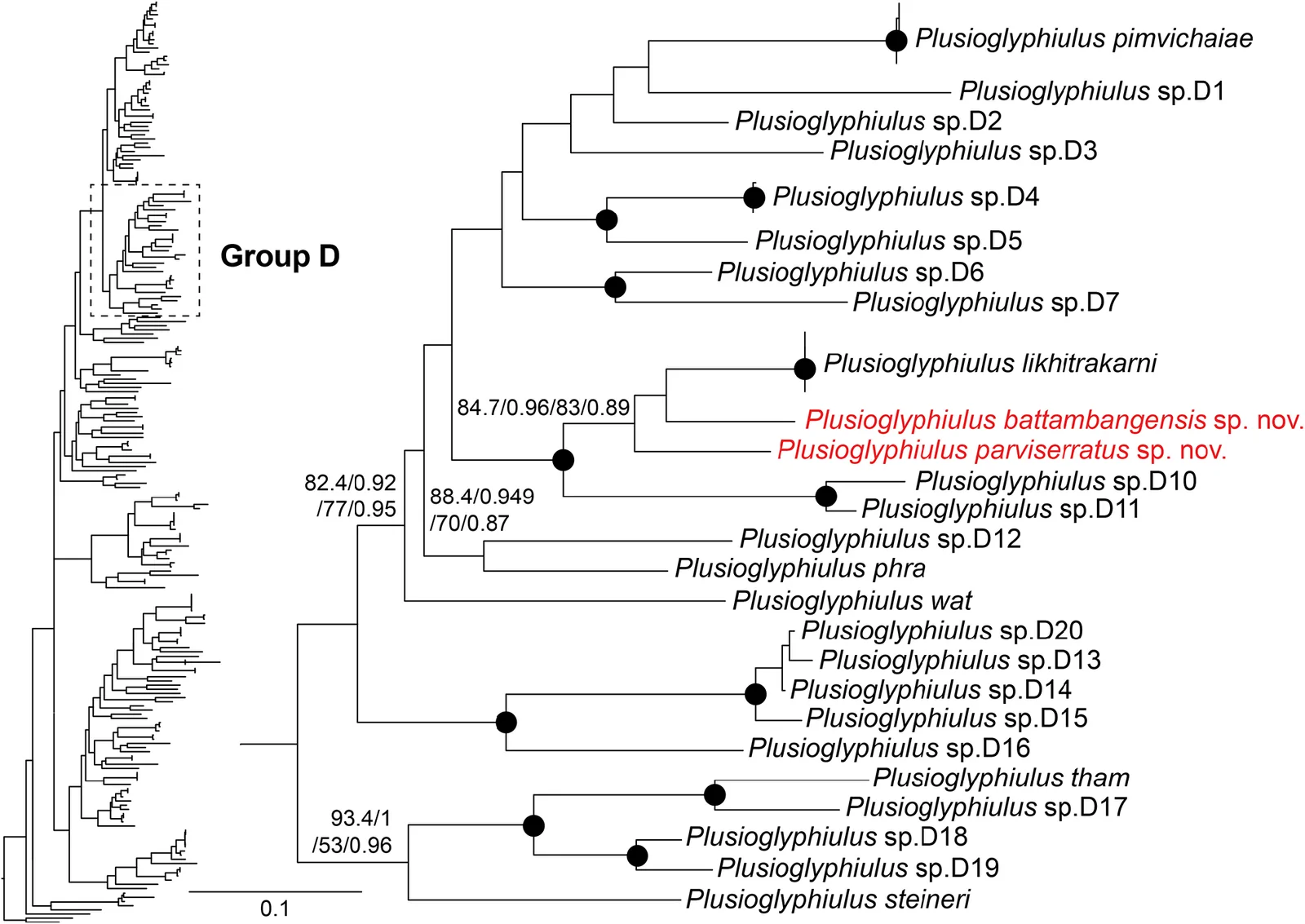
Molecular phylogeny of *Plusioglyphiulus* group D *sensu*Seesamut et al. (2026) indicating the positions of *P. battambangensis* sp. nov. and *P. parviserratus* sp. nov. Nodal support values are given as SH-aLRT/aBayes/ultra-fast bootstrap values from the maximum likelihood analysis in IQ-TREE, followed by posterior probabilities from the Bayesian inference analysis in MrBayes. A black circle on a node indicates a well-supported clade, with SH-aLRT ≥ 80%, aBayes ≥ 0.95, BS ≥ 95%, and PP ≥ 0.95.

The phylogenetic framework and species-group assignment followed Seesamut et al. (2026). In the present study, molecular data were used primarily to evaluate the genetic divergence of the two new species from their closest congeners, rather than to reconstruct an independent *de novo* phylogeny (Fig. 1). COI sequences of *Plusioglyphiulus* group D *sensu*Seesamut et al. (2026) were retrieved from GenBank to calculate uncorrected pairwise genetic distances (p-distances) using MEGA X (Kumar et al. 2018). Pairwise distances were estimated among the examined taxa, including the new species and its congeners, using the partial deletion option with a site coverage cut-off of 95%. The resulting p-distance values were used to assess the level of genetic divergence among taxa and to provide additional support for species delimitation in combination with morphological evidence.

## Results

The cave-dwelling millipedes of the genus *Plusioglyphiulus* in Cambodia currently comprise six species, including two new species described herein. The phylogenetic placement and species-group assignment of the two new species follow the COI-based framework of Seesamut et al. (2026), rather than representing an independent *de novo* phylogenetic reconstruction in the present study. Under this framework, both new species are assigned to Group D, which includes 26 species-level lineages.

Within Group D, the two new Cambodian species are positioned among closely related congeners but are genetically differentiated from their nearest lineages. Uncorrected pairwise COI p-distances within Group D ranged from 0.9% to 20.5% (Table 1). The two new Cambodian species were separated from their closest congeners by minimum interspecific distances of 9.6% and 9.9%, respectively. Although these values represent the lower range of interspecific divergence observed within the group, they are accompanied by clear diagnostic differences in morphology. The COI data are therefore congruent with the morphological evidence and provide supplementary support for the delimitation of the two new species.

**Table 1.** Matrix of the average uncorrected *p*-distance (%) based on 669-bp COI barcoding region.

**Taxa**	**1**	**2**	**3**	**4**	**5**	**6**	**7**	**8**	**9**	**10**	**11**	**12**	**13**	**14**	**15**	**16**	**17**	**18**	**19**	**20**	**21**	**22**	**23**	**24**	**25**
*Plusioglyphiulus* sp. D10																									
***Plusioglyphiulus parviserratus* sp. nov**.	0.139																								
***Plusioglyphiulus battambangensis* sp. nov**.	0.145	0.103																							
*Plusioglyphiulus* sp. D3	0.160	0.146	0.143																						
*Plusioglyphiulus* sp. D12	0.152	0.132	0.138	0.155																					
*Plusioglyphiulus likhitrakarni*	0.138	0.099	0.096	0.155	0.151																				
*Plusioglyphiulus tham*	0.178	0.179	0.181	0.170	0.188	0.184																			
*Plusioglyphiulus pimvichaiae*	0.155	0.121	0.152	0.146	0.149	0.146	0.194																		
*Plusioglyphiulus* sp. D4	0.160	0.139	0.149	0.121	0.136	0.157	0.179	0.129																	
*Plusioglyphiulus* sp. D1	0.163	0.145	0.138	0.145	0.157	0.149	0.200	0.149	0.163																
*Plusioglyphiulus* sp. D18	0.178	0.155	0.158	0.164	0.160	0.176	0.133	0.178	0.173	0.169															
*Plusioglyphiulus* sp. D14	0.190	0.166	0.170	0.188	0.170	0.182	0.190	0.172	0.167	0.193	0.179														
*Plusioglyphiulus phra*	0.155	0.135	0.138	0.139	0.123	0.152	0.170	0.152	0.127	0.143	0.148	0.161													
*Plusioglyphiulus* sp. D6	0.160	0.154	0.143	0.146	0.157	0.145	0.181	0.151	0.130	0.145	0.161	0.175	0.136												
*Plusioglyphiulus* sp. D16	0.172	0.170	0.161	0.173	0.163	0.164	0.181	0.160	0.173	0.164	0.175	0.155	0.164	0.149											
*Plusioglyphiulus* sp. D7	0.164	0.151	0.164	0.161	0.170	0.167	0.205	0.164	0.158	0.155	0.188	0.155	0.155	0.115	0.164										
*Plusioglyphiulus* sp. D15	0.191	0.169	0.176	0.193	0.170	0.188	0.187	0.173	0.161	0.203	0.185	0.040	0.167	0.179	0.167	0.169									
*Plusioglyphiulus* sp. D20	0.188	0.161	0.167	0.190	0.170	0.181	0.187	0.169	0.167	0.190	0.175	0.009	0.158	0.176	0.152	0.157	0.046								
*Plusioglyphiulus* sp. D13	0.193	0.169	0.170	0.197	0.176	0.187	0.194	0.170	0.170	0.191	0.184	0.019	0.167	0.182	0.160	0.161	0.054	0.016							
*Plusioglyphiulus* sp. D5	0.149	0.154	0.142	0.130	0.145	0.143	0.176	0.138	0.103	0.154	0.164	0.161	0.149	0.130	0.148	0.155	0.166	0.163	0.161						
*Plusioglyphiulus* sp. D11	0.051	0.132	0.141	0.146	0.152	0.133	0.181	0.152	0.149	0.158	0.182	0.188	0.161	0.158	0.176	0.161	0.184	0.187	0.191	0.158					
*Plusioglyphiulus* sp. D2	0.151	0.142	0.142	0.118	0.139	0.123	0.182	0.127	0.111	0.138	0.163	0.182	0.146	0.117	0.160	0.143	0.178	0.182	0.182	0.127	0.142				
*Plusioglyphiulus* sp. D19	0.172	0.157	0.160	0.157	0.175	0.176	0.130	0.170	0.170	0.179	0.054	0.188	0.151	0.169	0.181	0.187	0.196	0.184	0.187	0.172	0.172	0.172			
*Plusioglyphiulus wat*	0.160	0.138	0.148	0.155	0.158	0.154	0.185	0.146	0.151	0.146	0.178	0.161	0.148	0.160	0.182	0.178	0.164	0.160	0.164	0.143	0.158	0.155	0.175		
*Plusioglyphiulus steineri*	0.166	0.148	0.154	0.172	0.157	0.167	0.164	0.169	0.175	0.166	0.143	0.167	0.157	0.158	0.166	0.160	0.167	0.164	0.170	0.175	0.166	0.161	0.157	0.152	
*Plusioglyphiulus* sp. D17	0.178	0.166	0.178	0.176	0.172	0.172	0.093	0.191	0.166	0.187	0.129	0.188	0.164	0.181	0.182	0.202	0.193	0.185	0.190	0.175	0.182	0.175	0.136	0.191	0.167

### Taxonomic account

#### Order Spirostreptida

**Family Cambalopsidae Cook, 1895**

**Genus *Plusioglyphiulus* Silvestri, 1923**

##### Plusioglyphiulus dubius

Taxon classificationAnimaliaSpirostreptidaCambalopsidae

(Attems, 1938)

D2FA8FC6-8B69-5950-9B30-524862264F99

Glyphiulus dubius Attems, 1938: 272 (D).
Plusioglyphiulus dubius —Mauriès 1970: 510 (M); 1983: 272 (D); Hoffman 1977: 715 (M); Jeekel 2004: 57 (R); Golovatch et al. 2009: 74 (R); 2011: 4 (L, M); Likhitrakarn et al. 2015: 179 (L); 2020a: 139 (L, M).

###### Remarks.

Attems (1938) originally described this species as *Glyphiulus dubius* based on a single female holotype obtained from Angkor, Cambodia. Mauriès (1983) subsequently noted a striking morphological resemblance between *P. dubius* and *P. boutini* Mauriès, 1970, distinguishing them primarily by the starting position of the divided dorsal crests (on body ring 6 in *P. dubius* vs ring 7 in *P. boutini*). However, because the diagnostic male material remains entirely unavailable, the definitive verification of this species is severely hindered. In the complete absence of male gonopod structures, which are critical for accurate generic and species placement, our current diagnosis must rely exclusively on overall somatic characters and body shape parameters. Until male topotypes are collected to clarify its true identity, *P. dubius* remains a species of doubtful status within the Cambodian fauna.

##### Plusioglyphiulus boutini

Taxon classificationAnimaliaSpirostreptidaCambalopsidae

Mauriès, 1970

6B651AF8-1F0B-53DD-B684-07C78E89281A

Plusioglyphiulus boutini Mauriès, 1970: 509.
Plusioglyphiulus boutini —Hoffman 1977: 715; Mauriès 1983: 272; Boutin 2001: 1760; Jeekel 2004: 57; Golovatch et al. 2009: 72; 2011: 4; Likhitrakarn et al. 2015: 179; 2020a: 139 (R, D, M).

###### Remarks.

This species was originally described from a cave ecosystem near Kampong Trach, Kampot Province, Cambodia (Mauriès 1970). The newly accumulated material documented herein was collected from Phnom Kampong Trach Cave and Prasat Phnom Totong Temple, located only ~ 17 km away from the type locality. As illustrated in our updated distribution map (Fig. 12), these new sampling sites are remarkably close to each other, confirming that the new specimens collected by Likhitrakarn et al. (2020a) represent highly localized populations in immediate geographical proximity. The morphology of the newly examined specimens fully agrees with the original description and details provided by Mauriès (1970).

##### Plusioglyphiulus biserratus

Taxon classificationAnimaliaSpirostreptidaCambalopsidae

Likhitrakarn, Golovatch, Thach, Chhuoy, Ngor, Srisonchai, Sutcharit & Panha, 2020

21DA0F5B-EE17-501A-ADB9-97CCE42254B5

Plusioglyphiulus biserratus Likhitrakarn et al., 2020a: 141 (D).

###### Remarks.

This distinct cavernicolous species was originally described from Phnom Kbal Romeas Cave in Tuek Chhou District, Kampot Province, southern Cambodia (Likhitrakarn et al. 2020a). Based on topotypic material, *P. biserratus* is confirmed as a narrow endemic species strictly confined to its isolated type locality, underscoring a pronounced pattern of cave micro-endemism within the fragmented karst towers of Indochina. Despite its strict localization, the diagnostic features of this species remain exceptionally stable across all examined populations, standing out sharply from all other known Cambodian congeners by its unique male gonopods, particularly the simplified, single-structured anterior processes and the distinctly serrate apicolateral margins of the posterior gonopodal telopodites.

##### Plusioglyphiulus khmer

Taxon classificationAnimaliaSpirostreptidaCambalopsidae

Likhitrakarn, Golovatch, Thach, Chhuoy, Ngor, Srisonchai, Sutcharit & Panha, 2020

FCC04BA6-6CA7-542F-A07D-1B422C0ACB36

Plusioglyphiulus khmer Likhitrakarn et al., 2020a: 144 (D).

###### Remarks.

This remarkable species was originally described from Phnom Sorsia Temple Cave in Damnak Chang’aeur District, Kep Province, southern Cambodia (Likhitrakarn et al. 2020a). The morphology of *P. khmer* is distinctive within the genus, being readily distinguished from all other known Cambodian congeners by its complete and entirely undivided longitudinal crests on the collum. This conspicuous configuration provides a strong diagnostic contrast to the regional congeners, which always show divided and incomplete crests. Furthermore, the species is characterized by a complex arrangement of long, slender coxosternal processes (cxp1 and cxp2) of the anterior gonopods.

##### Plusioglyphiulus parviserratus

Taxon classificationAnimaliaSpirostreptidaCambalopsidae

Likhitrakarn & Seesamut sp. nov.

52245CC1-46C4-5D82-BA7A-00A5C1751D16

https://zoobank.org/AE7C6DD1-F369-4748-A9F6-27C11ED7C5A1

Figs 2A, 3–7

Plusioglyphiulus sp. D9: Seesamut et al. 2026: 9 (MI).

###### Type material.

***Holotype***. • ♂ (CUMZ-CAM236), Cambodia, Battambang Province, Banan District, Phum Thmei, Wat La-ang Phnom Takream (locality code C104), 13°04'27"N, 103°03'32"E, 01.08.2024, leg. C. Sutcharit and R. Srisonchai. ***Paratypes***. • 26 ♂, 43 ♀ (CUMZ-CAM236), same locality, together with holotype.

**Figure 2.**
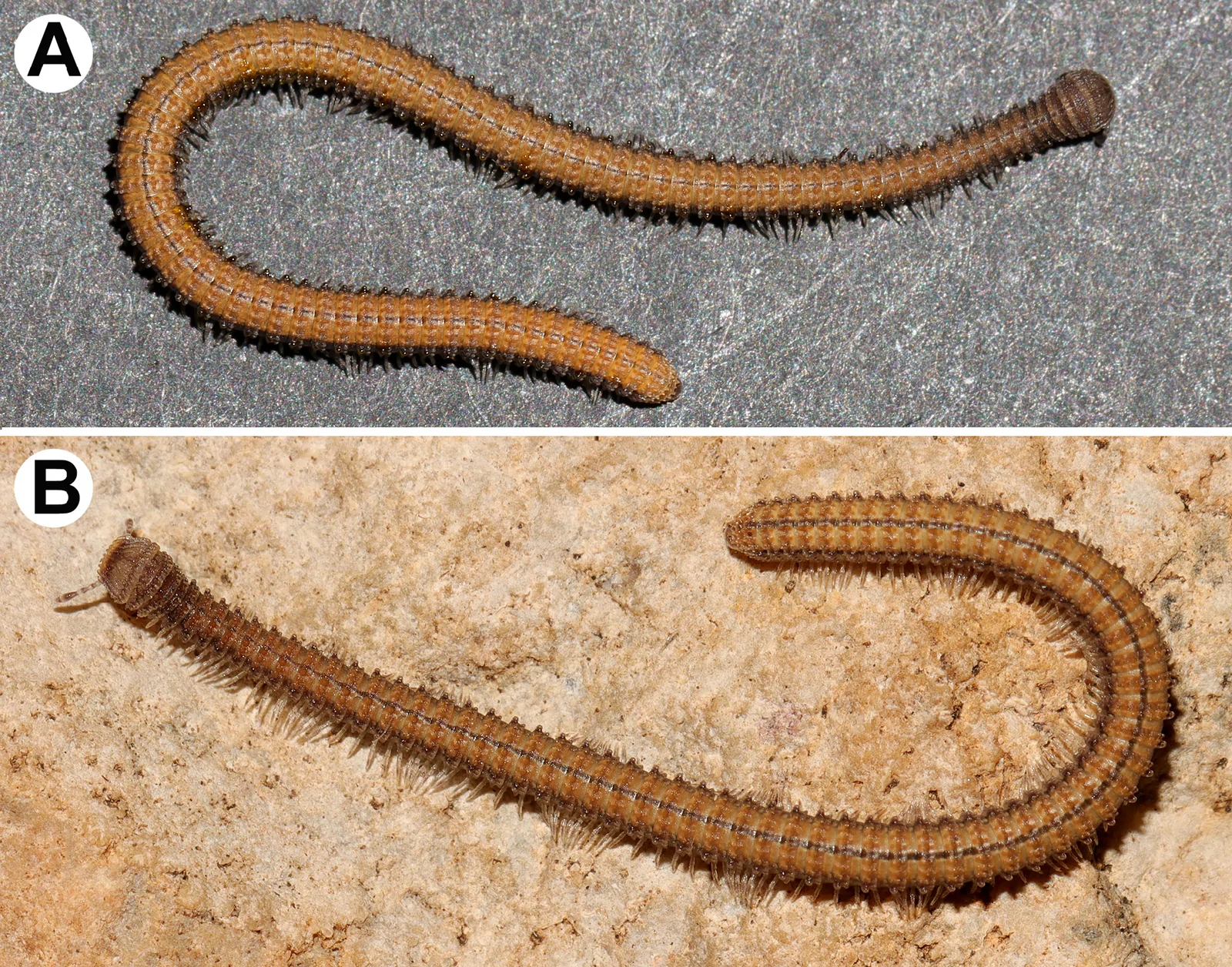
Habitus, live coloration. **A**. *Plusioglyphiulus parviserratus* sp. nov., ♂ paratype; **B**. *Plusioglyphiulus battambangensis* sp. nov., ♀ paratype. All pictures by R. Srisonchai, not taken to scale.

###### Diagnosis.

**Figure 3.**
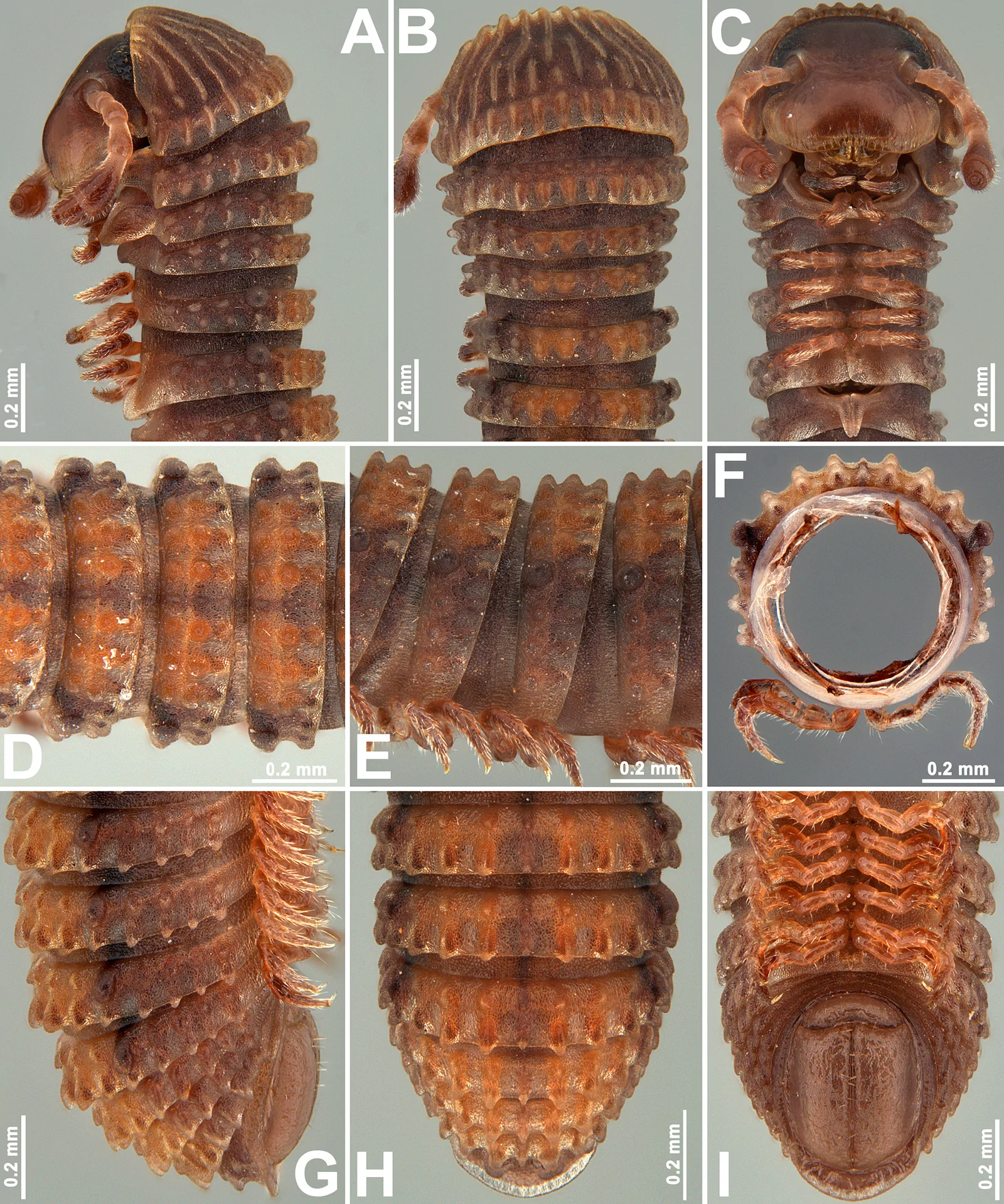
*Plusioglyphiulus parviserratus* sp. nov., ♂ paratype. **A–C**. Anterior part of body, lateral, dorsal, and ventral views, respectively; **D, E**. Midbody segments, dorsal and lateral views, respectively; **F**. Cross-section of a midbody segment; **G–I**. Posterior part of body, lateral, dorsal, and ventral views, respectively.

**Figure 4.**
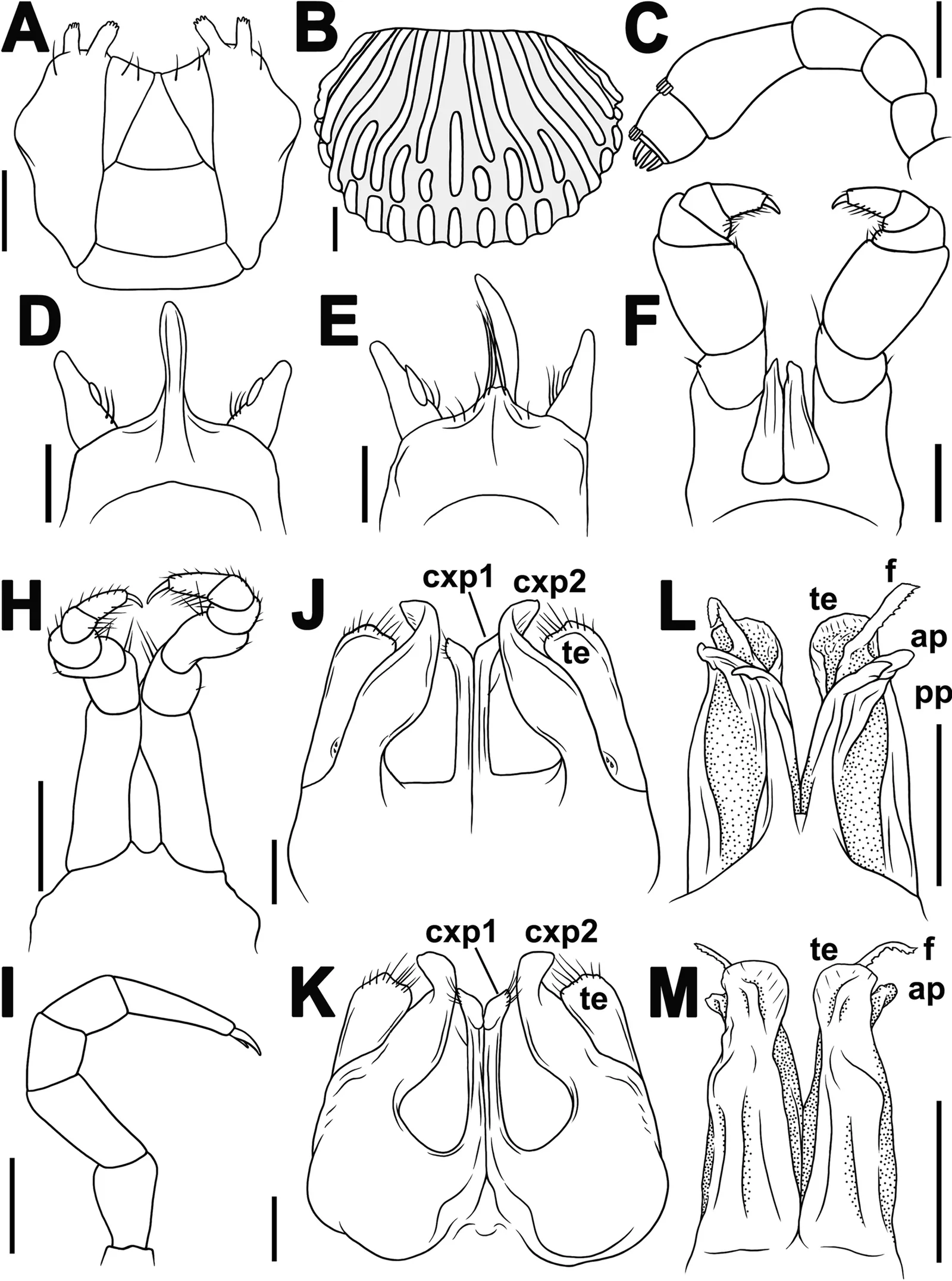
*Plusioglyphiulus parviserratus* sp. nov., ♂ holotype. **A**. Gnathochilarium, ventral view; **B**. Collum, dorsal view; **C**. Antenna, lateral view; **D, E**. ♂ legs 1, anterior and posterior views, respectively; **F**. ♂ legs 2, posterior view; **G**. ♂ legs 3, posterior view; **H**. Midbody rings, ventral view; **I, J**. Anterior gonopods, posterior and anterior views, respectively; **K, L**. Posterior gonopods, posterior and anterior views, respectively. Abbreviations: ap = anterior coxal process, cxp 1 = anterior coxosternal processes, cxp 2 = posterior coxosternal processes, f = flagellum process, pp = paramedian coxal process, te = telopodite. Scale bars: 0.1 mm.

**Figure 5.**
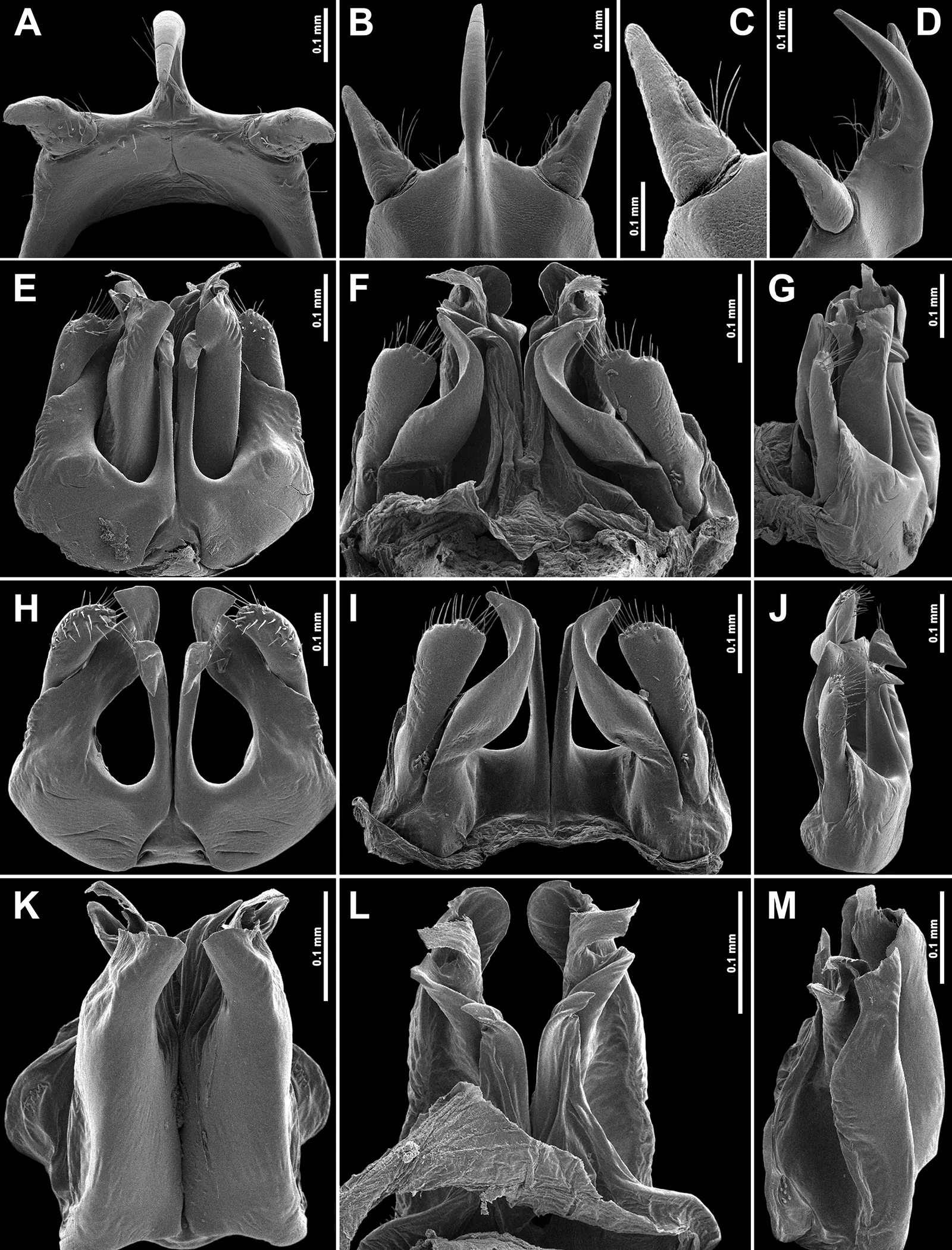
*Plusioglyphiulus parviserratus* sp. nov., ♂ holotype (**A–C, H–M**), ♂ paratype (**E–G**). **A, B, D**. Legs 1, subcaudal, frontal and sublateral views, respectively; **C**. Leg 1, frontal view; **E–G**. Anterior and posterior gonopods, anterior, subposterior and sublateral views, respectively; **H–J**. Anterior gonopods, frontal, caudal and lateral views, respectively; **K–M**. Posterior gonopods, subfrontal, caudal and lateral views, respectively.

**Figure 6.**
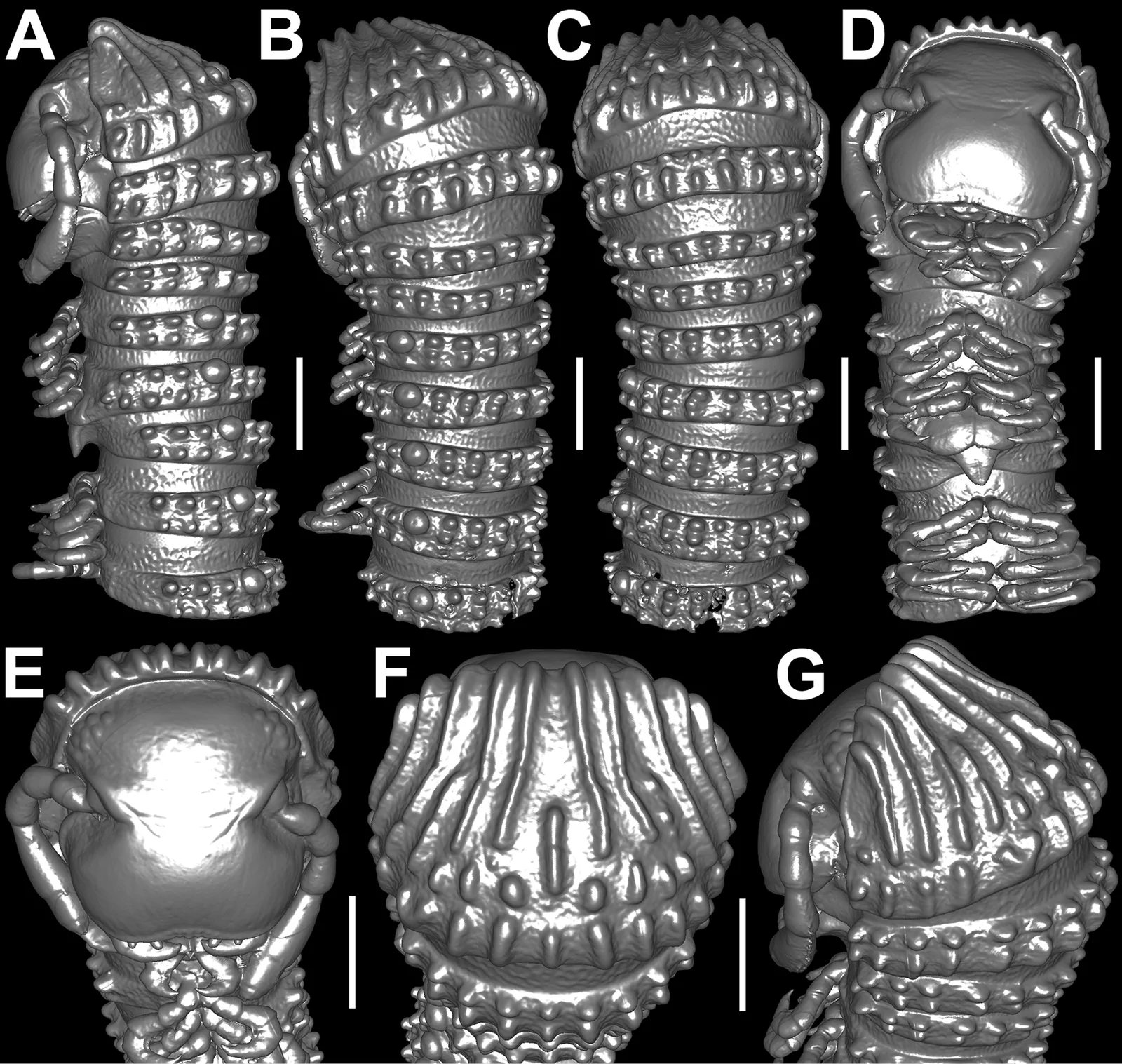
*Plusioglyphiulus parviserratus* sp. nov., ♂ male paratype, shaded surface display volume renderings based on 3DµCT models. **A–D**. Anterior part of body, lateral, sublateral, dorsal and ventral views, respectively; **E**. Head and anterior part of body, ventral view; **F, G**. Collum and anterior part of body, dorsal and lateral views, respectively. Scale bars: 0.1 mm.

This new species seems to be especially closely related to *Plusioglyphiulus likhitrakarni* Golovatch, Geoffroy, Mauriès & VandenSpiegel, 2011, a species described from eastern Thailand, sharing with the latter congener the highly similar general somatic characters and gonopod configuration. Both species, however, can be distinguished by a lower number of ommatidia rows, each eye patch with only 7–16 ommatidia arranged in 2–3 longitudinal rows (Figs 3A, 3C, 6C–E, 6G) (vs 15–22 ocelli arranged in 5–6 longitudinal rows in *P. likhitrakarni*), and by the distinct carinotaxic formula of the collum: (1a)/t+2p/t+3(3p/t)+4(4p/t)+ta/t+(5p)/t/t+ta/t+pp/(t)/t+m/m (Figs 3A, 3B, 4B, 6A–C, 6F, 6G) (compared to //(t)+1p/t//t+/(t)/t+2p//t+/(t)/t+3p//(t)/t+/(t)/t+4p/(t)/t+pp/t (or pp/(t)/t)+/ma/ma/m in *P. likhitrakarni*). They also differ in the carinotaxy of the midbody metaterga, with the formula of metatergum 5 and subsequent ones, except for the last few rings, usually following the pattern of 3/3+I/i+3/3/3+m/m (Figs 3A, 3B, 3D–H, 6A–C) (as opposed to 4/4+I/i+3/3/3+m/m in *P. likhitrakarni*). Furthermore, the telopodites of male legs 1 are distinctly 2-segmented (Figs 4D, 4E, 5A–D) (vs 1-segmented in *P. likhitrakarni*), and the anterior coxosternal processes (cxp1) of the anterior gonopods are regularly curved anteriorly, vividly resembling a bird’s head (Figs 4I, 4J, 5E–J), whereas they are characteristically contiguous and digitiform in *P. likhitrakarni*.

###### Description.

Length of holotype, 28.2 mm; of adult paratypes, 19.4–35.3 mm (♂) or 20.5–40.4 mm (♀); midbody rings round in cross-section (Fig. 3F), their width (horizontal diameter) and height (vertical diameter) similar, width in holotype 1.6 mm; paratypes 1.3–1.8 mm (♂) or 1.6–1.9 mm (♀).

***Coloration*** of live animals light brown (Fig. 2A), with darker lateral sides and a narrow median line; head and anterior body rings (rings 1–6) usually darker brownish; antennae, venter, and legs light yellowish (Fig. 2A). Coloration in alcohol, after two years of preservation (Fig. 3A–I), fading to uniformly reddish brown or dark castaneous brown to grey-brown, with the median line, porosteles, and surrounding areas usually dark brownish (Fig. 3A, B, D, E, G, H); antennae and venter yellow-brownish to brownish (Fig. 3A–C, E–G, I); eyes dark brown to blackish (Fig. 3A, C).

***Adult body*** with 50p+4a+T (holotype); paratypes with 53–74p+1–5a+T (♂) or 54–79p+2–3a+T (♀). Eye patches transversely ovoid, with 7–16 flat ommatidia arranged in 2–3 longitudinal rows (Figs 3A, 3C, 6A, 6D, 6E, 6G). Clypeus with three teeth anteromedially (Figs 3C, 6D, 6E).

***Antennae*** short and clavate (Figs 3A–C, 4C, 6A, 6D, 6E, 6G), extending to ring 2 laterally, antennomeres 5 and 6 each with a small apicodorsal field or corolla of bacilliform sensilla (Fig. 4C). Gnathochilarium oligotrichous, each lamella lingualis with 2–3 setae; promentum bare, separated from eumentum by a distinct transverse suture (*n* = 2) (Fig. 4A).

***Postcollum constriction*** evident, but collum moderately enlarged (Figs 2A, 3B, 3C, 6A–F). Carinotaxic formula of collum: (1a)/t+2p/t+3(3p/t)+4(4p/t)+ta/t+(5p)/t/t+ta/t+pp/(t)/t+ma/m (Figs 3A, 3B, 4B, 6A–C, 6F, 6G). Carinotaxy of metatergum 2: 8/8+m/m+8/8; metatergum 3: 8/7+m/m+8/7; metatergum 4: 7/7+m/m+7/7 (Figs 3A, 3B, 6A–C, 6F, 6G). Metatergal formula 5 and subsequent ones, except for the last few rings, usually 3/3+I/i+3/3/3+m/m+3/3/3+I/i+3/3 (Figs 3A, 3B, 3D–H, 6A–C); that of legless rings usually 7+m+7 (Fig. 5G, H). All crests and tubercles rather low (Figs 3A, 3B, 3D–H, 6A–C, 6F, 6G). Porosteles large, low, conical, rounded, directed anterolaterad, and higher than wide (Figs 3A, 3B, 3D–H, 6A–C). Midbody rings subcircular in cross-section, virtually uncompressed laterally (Fig. 3F).

***Tegument*** finely alveolate-areolate (Figs 3A, 3B, 3D, 3E, 3G, 3H, 6A–C, 6F, 6G), dull throughout. Metatergal setae absent. Pleural regions of rings 2–4 elongated, flap-shaped, especially prominent on ring 3 (Figs 3A, 3C, 6A, 6D).

***Epiproct*** (Fig. 3G, H) broadly rounded apically, with 2+2 paramedian tubercles, the median tubercles larger than the lateral ones. Paraprocts smooth, regularly convex, and densely setose (Fig. 3G, I). Hypoproct transversely bean-shaped, concave posteriorly (Fig. 3I).

***Ventral flaps*** behind gonopod aperture on male ring 7 evident (Figs 3A, 3C, 6A, 6D), distinguishable as low swellings with rounded lobes bent abruptly posteriorly.

***Legs*** short, ~ 2/3 as long as body diameter (Figs 3F, 4H); claw with a strong, spiniform, accessory claw at base, the latter almost half as long as claw itself (Fig. 4H).

**Figure 7.**
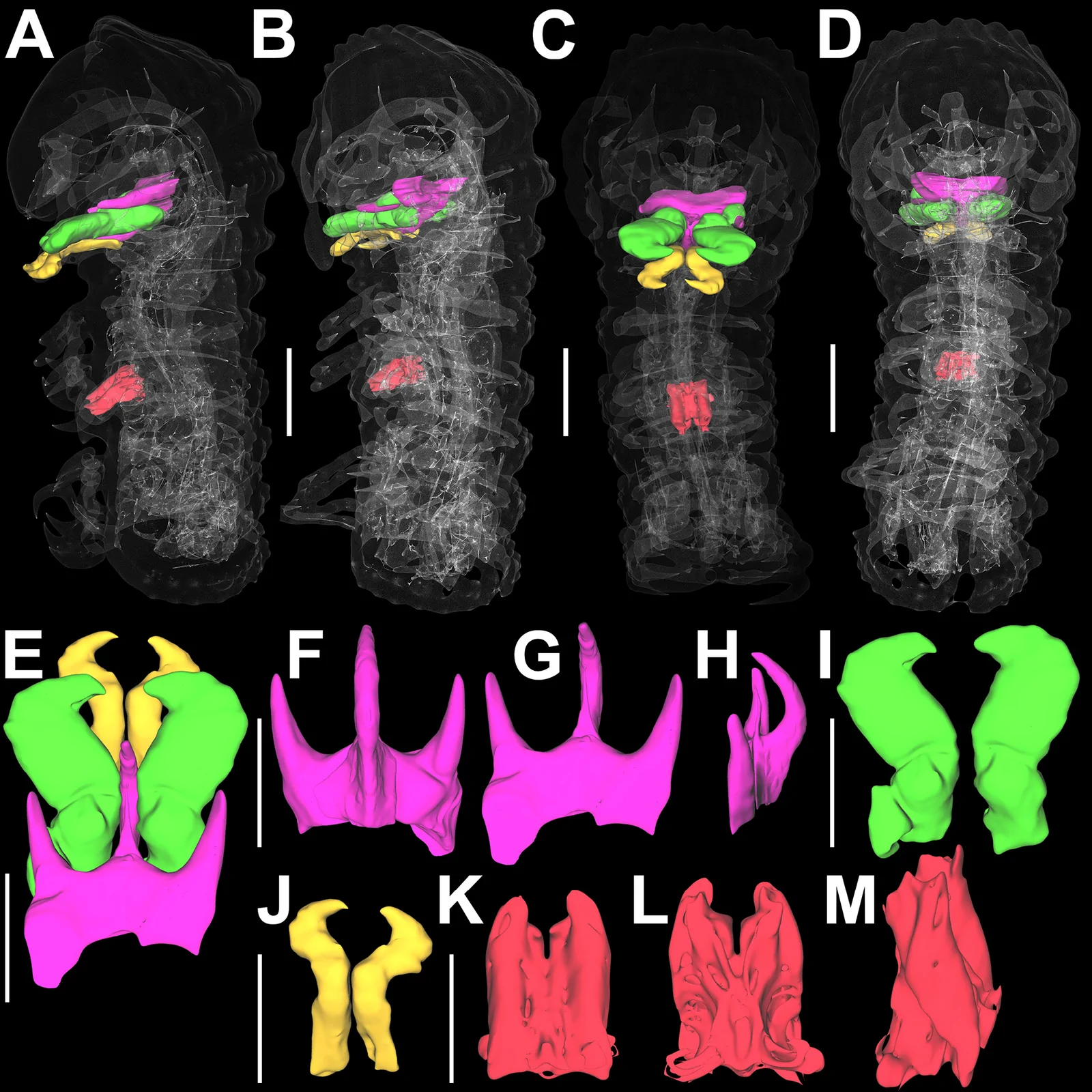
Shaded surface display volume renderings from 3DµCT data showing the diagnostic character matrix of *Plusioglyphiulus parviserratus* sp. nov., male paratype. **A–D**. Anterior part of body in lateral, sublateral, dorsal, and ventral views, respectively (the internal structures exposed via virtual dissection). Distinct anatomical components are schematically color-coded to visualize structural boundaries: leg 1 (purple), leg 2 (green), leg 3 (yellow), and the anterior and posterior gonopods (red); **E**. Legs 1–3 *in situ*, anterior view; **F–H**. Legs 1, posterior, anterior, and lateral views, respectively; **I**. Leg 2, anterior view; **J**. Leg 3, anterior view; **K–M**. Anterior and posterior gonopods, anterior, posterior, and sublateral views, respectively. Scale bars: 0.1 mm.

***Male legs 1*** with a usual, strong, and long central hook (actually a pair of tightly appressed hooks) regularly curved anteriorly; a pair of strong, sac-shaped, 2-segmented telopodites nearly half as long as central hook (Figs 4D, 4E, 5A–D, 7E–H), with a small second telopoditomere retained as a rudimentary knob (Figs 4D, 4E, 5A–C).

***Male legs 2*** strongly enlarged, with high and large coxae; telopodites hirsute on anterior face; penes broad, oblong-subtrapeziform, fused at base (Figs 4F, 6D, 7E, 7I).

***Male legs 3*** modified as usual, with particularly elongate and slender coxae, and shortened telopodites (Figs 4G, 6D, 7E, 7J).

***Anterior gonopods*** (Figs 4I, 4J, 5E–J) rather complex; coxosternal halves in contact, but not completely fused medially (Figs 4I, 4J, 5E, 5H, 5I). Anterior coxosternal processes (cxp1) forming a paramedian pair, long, slender, erect, setose only apically, and regularly curved anteriorly, resembling a bird’s head (Figs 4J, 5E, 5G, 5H, 5J). Posterior coxosternal processes (cxp2) higher, distally lamellate, with the apices rounded and turned laterally (Figs 4I, 4J, 5F, 5H, 5I). Telopodites (te) club-shaped, subcylindrical, movable, 1-segmented, apically setose, and shorter than cxp2 (Figs 4I, 4J, 5E–J).

***Posterior gonopods*** (Figs 4K, 4L, 5E–G, 5K–M) compact, shorter; coxosternum well separated from sternum, fused only basally. Each coxite with an evident, stout, paramedian coxal process (pp) acute distally and directed laterally (Figs 4K, 5F, 5K–M). Anterior coxal process (ap) lamellate, subquadrate (Figs 4K, 4L, 5E–G, 5K–M), bearing a high, broad flagellum process (f) with the apex distinctly revolute laterally and a micro-serrate upper margin. (Figs 4K, 4L, 5E–G, 5K–M). Telopodite (te) elongate, membranous, apically rounded, with lateral margins slightly rugose, distinctly higher than both pp and ap (Figs 4K, 4L, 5E–G, 5K–M).

###### Habitat.

The specimens were collected in the dark zone of the cave, exclusively on bat guano deposits. The cave environment maintains high humidity and stable ambient temperatures throughout the year, characteristic of the limestone karst systems in Battambang Province, Cambodia.

###### Etymology.

The specific epithet is a Latinized adjective derived from a combination of the Latin prefix *parvi*- (meaning small) and the adjective *serratus* (meaning saw-like), highlighting the distinct micro-serrate upper margin of the broad flagellum process (f) of the posterior gonopods; adjective.

###### Remarks.

Morphologically, *Plusioglyphiulus parviserratus* sp. nov. seems to be especially similar to *P. likhitrakarni*, a congener originally described from eastern Thailand, with which it shares a remarkably similar general somatic characters and the basic structure of the gonopods. However, the new species is readily distinguishable from *P. likhitrakarni* by its unique carinotaxic pattern on the collum and midbody metaterga, as well as a significantly reduced number of ommatidia.

This sharp morphological differentiation is robustly supported by molecular data; the uncorrected pairwise *p*-distance between *P. parviserratus* sp. nov. and *P. likhitrakarni* at the COI barcode locus yields a significant divergence of 9.9% (Table 1). Although this value closely approaches the lower threshold of the typical interspecific distances separating valid congeners within *Plusioglyphiulus* (which generally range between 11.4% and 18.2%, with a mean interspecific divergence of 14.5%–15.1% depending on the species group (Seesamut et al. 2026)), such a substantial genetic gap coupled with the diagnostic boundaries fully corroborates the independent evolutionary status of the newly described taxon.

##### Plusioglyphiulus battambangensis

Taxon classificationAnimaliaSpirostreptidaCambalopsidae

Likhitrakarn & Seesamut sp. nov.

D8533F7D-85E3-544A-B001-0E112D6221FB

https://zoobank.org/AC99FBFA-A37B-4580-BB94-FDECF2945F6D

Figs 2B, 8–11

Plusioglyphiulus sp. D8: Seesamut et al. 2026: 9 (MI).

###### Type material.

***Holotype***. • ♂ (CUMZ-CAM237), Cambodia, Battambang Province, Banan District, Phumi Thnong, Asram Kompir Temple (locality code C105), 13°05'44"N, 102°55'32"E, 01.08.2024, leg. C. Sutcharit and R. Srisonchai. ***Paratypes***. • 17 ♂, 35 ♀ (CUMZ-CAM237), same locality, together with holotype.

###### Diagnosis.

**Figure 8.**
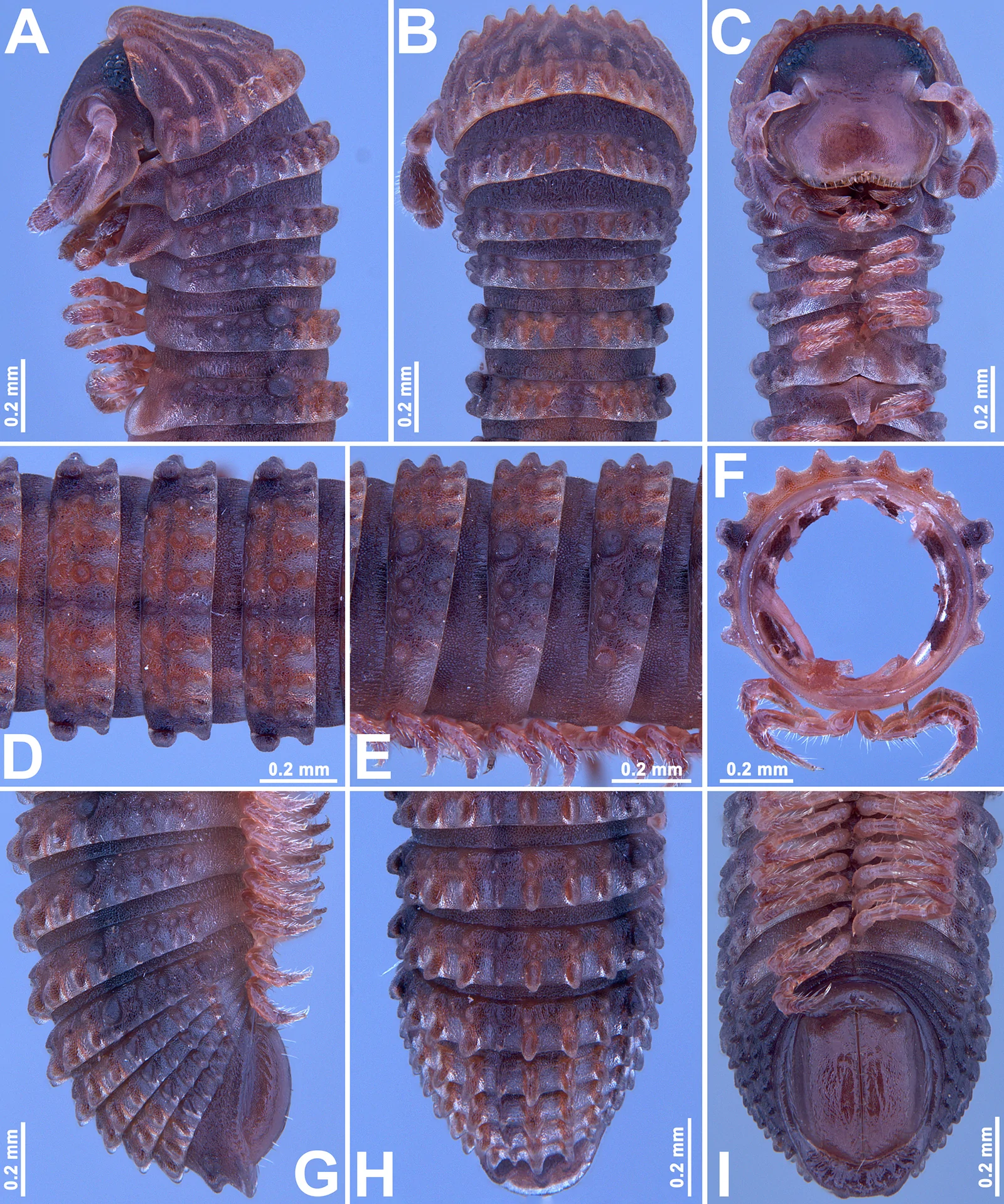
*Plusioglyphiulus battambangensis* sp. nov., ♂ paratype. **A–C**. Anterior part of body, lateral, dorsal, and ventral views, respectively; **D, E**. Midbody segments, dorsal and lateral views, respectively; **F**. Cross-section of a midbody segment; **G–I**. Posterior part of body, lateral, dorsal, and ventral views, respectively.

**Figure 9.**
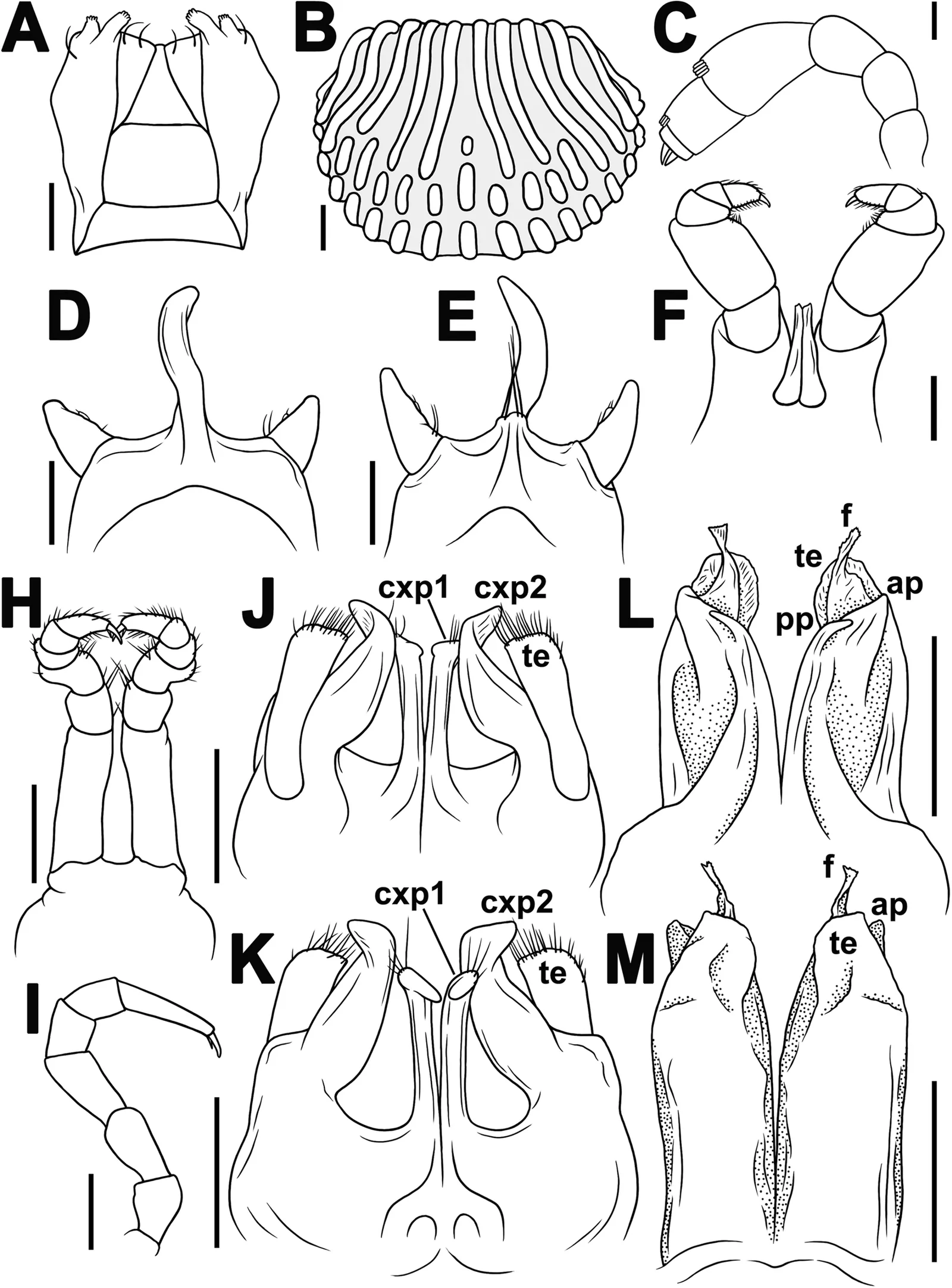
*Plusioglyphiulus battambangensis* sp. nov., ♂ paratype (**A, B**), ♂ holotype (**C–L**). **A**. Gnathochilarium, ventral view; **B**. Collum, dorsal view; **C**. Antenna, lateral view; **D, E**. ♂ legs 1, anterior and posterior views, respectively; **F**. ♂ legs 2, posterior view; **G**. ♂ legs 3, posterior view; **H**. Midbody rings, ventral view; **I, J**. Anterior gonopods, posterior and anterior views, respectively; **K, L**. Posterior gonopods, posterior and anterior views, respectively. Abbreviations: ap = anterior coxal process, cxp 1 = anterior coxosternal processes, cxp 2 = posterior coxosternal processes, f = flagellum process, pp = paramedian coxal process, te = telopodite. Scale bars: 0.1 mm.

**Figure 10.**
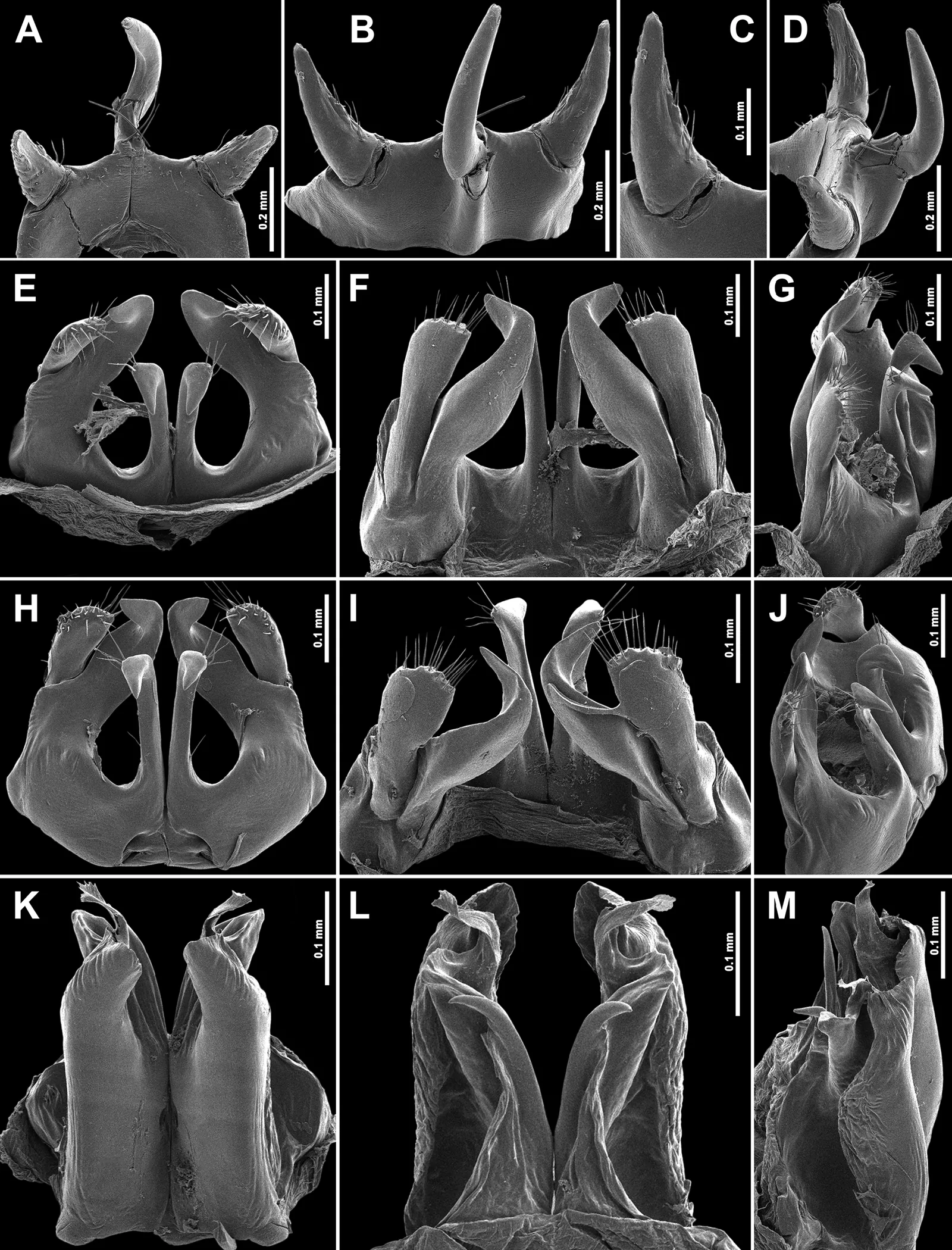
*Plusioglyphiulus battambangensis* sp. nov., ♂ holotype (**A–G, K–M**), ♂ paratype (**H–J**). **A, B, D**. Legs 1, caudal, subfrontal and sublateral views, respectively; **C**. Leg 1, subfrontal view; **E–J**. Anterior gonopods, subfrontal, caudal, sublateral, frontal, subcaudal and sublateral views, respectively; **K–M**. Posterior gonopods, frontal, caudal and sublateral views, respectively.

**Figure 11.**
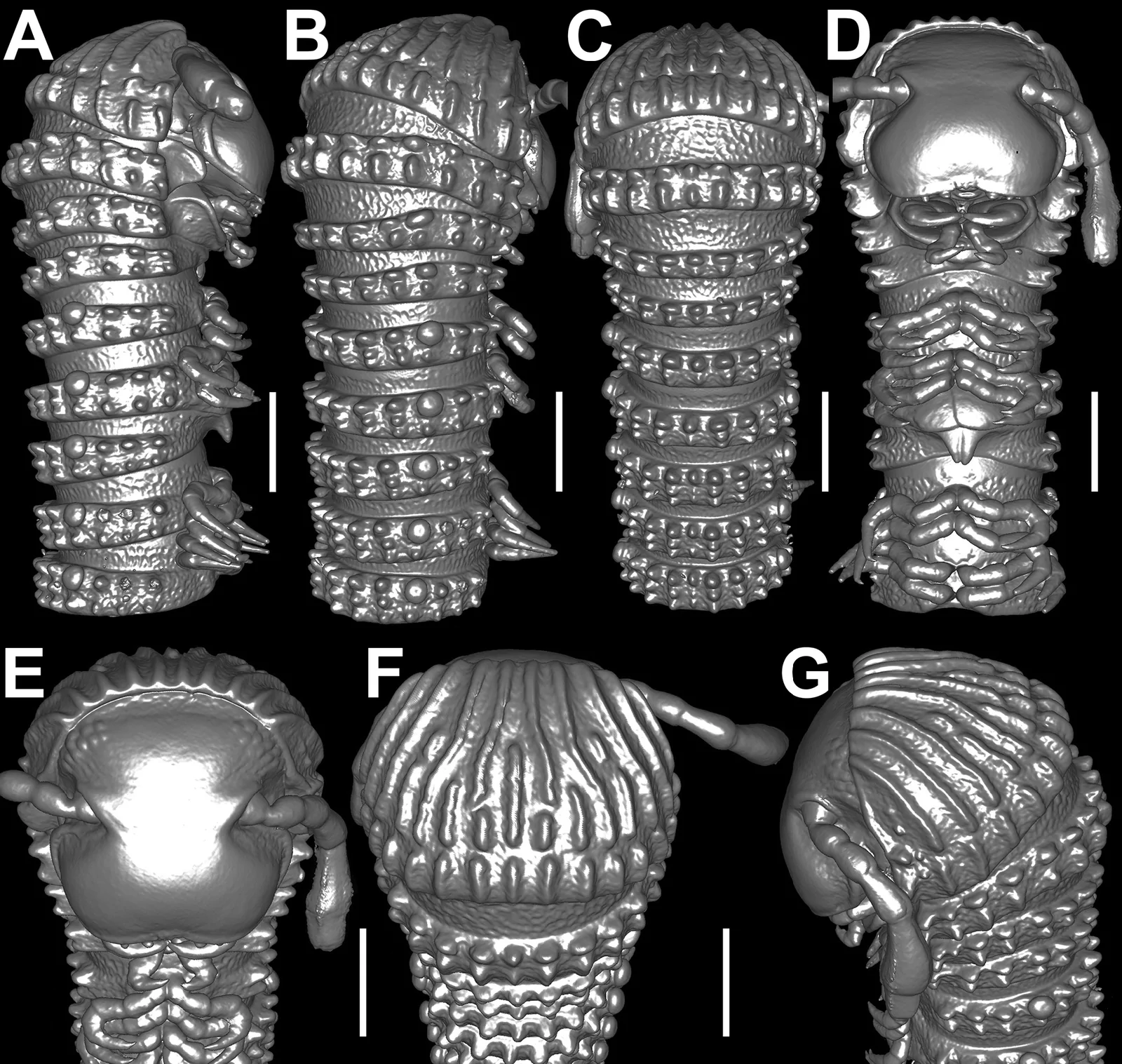
*Plusioglyphiulus battambangensis* sp. nov., ♂ male paratype, shaded surface display volume renderings based on 3DµCT models. **A–D**. Anterior part of body, lateral, sublateral, dorsal and ventral views, respectively; **E**. Head and anterior part of body, ventral view; **F, G**. Collum and anterior part of body, dorsal and lateral views, respectively. Scale bars: 0.1 mm.

This new species seems to be especially similar to *Plusioglyphiulus parviserratus* sp. nov. in both somatic characters and the structure of the posterior gonopods. Both species, however, can be distinguished by the carinotaxic formula of the collum: 1a/t+2p/t+3p/t/(t)+4p/(t)/t+(ta)/tc/t+5p/(t)/t+ta/t+pp/t/t+m/m (Figs 8A, 8B, 9B, 11A–C, 11F, 11G) (compared to (1a)/t+2p/t+3(3p/t)+4(4p/t)+ta/t+(5p)/t/t+ta/t+pp/(t)/t+m/m (Figs 3A, 3B, 4B, 6A–C, 6F, 6G)), and by having the small second telopoditomere on the male legs 1 significantly smaller (Figs 9D, 9E, 10A–C) (as opposed to larger (Figs 4D, 4E, 5A–C)). Additionally, the posterior gonopod of the new species displays an elongated and slender paramedian coxal process (pp) (Figs 9K, 10L, 10M), in contrast to the more stout one in *P. parviserratus* sp. nov. (Figs 4K, 5F, 5K–M), also showing a narrow flagellum process (f) provided with a smooth upper margin (Figs 9K, 9L, 10K–M) (vs broad and with a micro-serrate upper margin (Figs 4K, 4L, 5E–G, 5K–M) in *P. parviserratus* sp. nov.).

###### Description.

Length of holotype, 29.5 mm; adult paratypes 17.8–41.4 (♂) or 21.8–41.6 mm (♀) long; midbody rings round in cross-section (Fig. 8F), their width (horizontal diameter) shorter than height (vertical diameter), width in holotype 2.2 mm; in paratypes 1.3–1.7 (♂), 1.1–1.9 mm (♀).

***Coloration*** of live animals light brown (Fig. 2B), with darker lateral sides and a narrow median line; head and anterior body rings (rings 1–5) usually darker brownish; antennae, venter, and legs light yellowish (Fig. 2B). Coloration in alcohol, after two years of preservation (Fig. 8A–I), faded to uniformly reddish brown or dark castaneous brown to grey-brown, with the median line, porosteles, and surrounding areas usually dark brownish (Fig. 8A, B, D, E, G, H). Antennae and venter yellow-brownish to brownish (Fig. 8A–C, E–G, I). Eyes dark brown to blackish (Fig. 8A, C).

***Adult body*** with 65p+4a+T (holotype); paratypes with 53–79p+1–5a+T (♂) and 54–76p+2–6a+T (♀). Eye patches transversely ovoid, each provided with 8–12 flat ommatidia arranged in 2–3 longitudinal rows in adults (Figs 8A, 8C, 11D, 11E, 11G). Clypeus with three teeth anteromedially (Figs 8C, 11D).

***Antennae*** short and clavate (Figs 2B, 8A–C, 9C, 11A, 11D–F), extending to ring 2 laterally; antennomeres 5 and 6 each with a small apicodorsal field or corolla of bacilliform sensilla (Fig. 9C). Gnathochilarium oligotrichous, each lamella lingualis with 2–3 setae; promentum bare, separated from eumentum by a distinct transverse suture (*n* = 2) (Fig. 9A).

***Postcollum constriction*** evident, with the collum only moderately enlarged (Figs 2B, 8A–C, 11B–F). Carinotaxic formula of collum: 1a/t+2p/t+3p/t/(t)+4p/(t)/t+(ta)/tc/t+5p/(t)/t+ta/t+pp/t/t+ma/(ma)/m (Figs 8A, 8B, 9B, 11A–C, 11F, 11G). Carinotaxy of metatergum 2: 8/8+m/m+8/8; metatergum 3: 8/7+m/m+8/7; metatergum 4: 7/7+m/m+7/7 (Figs 8A, 8B, 11A–C, 11F, 11G). Metatergal formula 5 and subsequent ones, except for the last few rings, usually 3/3+I/i+3/3/3+m/m+3/3/3+I/i+3/3 (Figs 8A, 8B, 8D–H, 11A–C); that of legless rings usually 7+m+7 (Fig. 8G, H). All crests and tubercles rather low (Figs 8A, 8B, 8D–H, 11A–G). Porosteles large, low, conical, rounded, directed anterolaterad, and higher than wide (Figs 8D–H, 11A–C, 11G). Midbody rings subcircular in cross-section, virtually uncompressed laterally (Fig. 8F).

***Tegument*** finely alveolate-areolate (Figs 8A, 8B, 8D, 8E, 8G, 8H, 11A–C, 11F, 11G), dull throughout. Metatergal setae absent. Pleural regions of rings 2–4 elongated, flap-shaped, especially clearly so on ring 3 (Figs 8A, 8C, 11A, 11D).

***Epiproct*** (Fig. 8G–I) broadly rounded apically, with 2+2 paramedian tubercles, the inner pair being visibly larger than the outer ones. Paraprocts smooth, regularly convex, and densely setose (Fig. 8G, I). Hypoproct transversely bean-shaped, concave posteriorly (Fig. 8I).

***Ventral flaps*** behind gonopod aperture on male ring 7 evident (Figs 8A, 8C, 11A, 11D), distinguishable as low swellings with rounded lobes bent abruptly posteriorly.

***Legs*** short, ~ 2/3 as long as body diameter (Figs 8F, 9H); claw with a strong, spiniform accessory claw at base, the latter almost half as long as claw itself (Fig. 9H).

***Male legs 1*** with a usual, prominent, and elongate central hook (actually a pair of tightly appressed hooks) regularly curved anteriorly; a pair of strong, sac-shaped, 2-segmented telopodites nearly half as long as central hook (Figs 9D, 9E, 10A–D), with a small second telopoditomere retained as a rudimentary knob (Figs 9D, 9E, 10A–D).

***Male legs 2*** strongly incrassate, with high and large coxae; telopodites hirsute on anterior face; penes broad, oblong-subtrapeziform, fused at base (Figs 9F, 11D).

***Male legs 3*** modified as usual, with particularly elongate and slender coxae, and shortened telopodites (Figs 9G, 11D).

***Anterior gonopods*** (Figs 9I, 9J, 10E–J) rather complex; coxosternal halves in contact, but not completely fused medially (Figs 9I, 9J, 10E, 10F, 10H, 10I). Anterior coxosternal processes (cxp1) forming a paramedian pair, long, slender, erect, setose only apically, and regularly curved anteriorly, resembling a bird’s head (Figs 9J, 10E, 10G, 10H, 10J). Posterior coxosternal processes (cxp2) higher, distally lamellate, with the apices rounded and turned laterally (Figs 9I, 9J, 10E–J). Telopodites (te) club-shaped, subcylindrical, movable, 1-segmented, apically setose, and shorter than cxp2 (Figs 9I, 9J, 10E–J).

***Posterior gonopods*** (Figs 9K, 9L, 10K–M) compact, shorter; coxosternum well separated from sternum, fused only basally. Each coxite with a long, slender, paramedian coxal process (pp) acute distally and directed laterally (Figs 9K, 10L, 10M). Anterior coxal process (ap) lamellate, subquadrate (Figs 9K, 9L, 10K–M), with a high, narrow flagellum process (f) with a smooth upper margin at its apex, the apex itself being distinctly revolute laterally (Figs 9K, 9L, 10K–M). Telopodite (te) erect, elongate, membranous, apically rounded, with lateral margins slightly rugose, distinctly higher than both pp and ap (Figs 9K, 9L, 10K–M).

###### Habitat.

The specimens were collected in the aphotic zone of the cave, ~ 20 m from the entrance, occurring on moist limestone floor. The cave environment maintains high humidity and stable ambient temperatures throughout the year, characteristic of the limestone karst systems in Battambang Province.

###### Etymology.

The specific epithet is a Latinized adjective derived from the type locality, Battambang Province, Cambodia, where the type series was collected; adjective.

###### Remarks.

Morphologically, *Plusioglyphiulus battambangensis* sp. nov. is remarkably similar to its congener, *P. parviserratus* sp. nov. Both new species share an extremely uniform habitus, a strong reduction in the number of ommatidia, and highly similar gonopod structures. Such profound morphological conservatism frequently makes phenotypic discrimination between closely related cavernicolous congeners particularly difficult. However, despite these superficial similarities, *P. battambangensis* sp. nov. can be separated from *P. parviserratus* sp. nov. by the specific carinotaxic ornamentation of the collum, as well as by the significantly stouter processes of the gonopods, both anterior and posterior.

**Figure 12.**
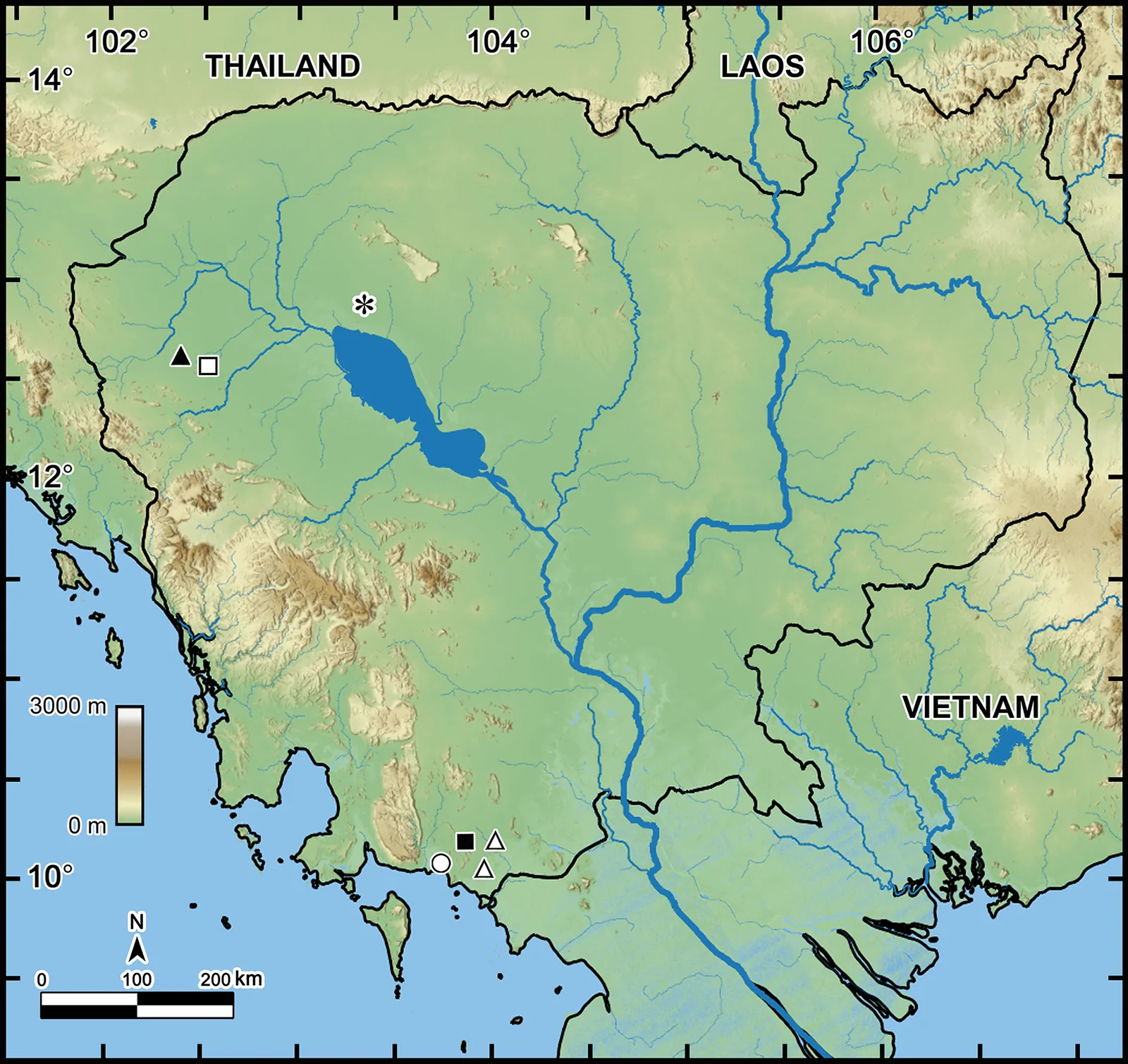
Distribution of *Plusioglyphiulus* species in Cambodia. Asterisk: *Plusioglyphiulus dubius* (Attems, 1938); filled triangle: *Plusioglyphiulus battambangensis* sp. nov.; open square: *Plusioglyphiulus parviserratus* sp. nov.; filled square: *Plusioglyphiulus khmer* Likhitrakarn, Golovatch, Thach, Chhuoy, Ngor, Srisonchai, Sutcharit & Panha, 2020; open triangle: *Plusioglyphiulus boutini* Mauriès, 1970; open circle: *Plusioglyphiulus biserratus* Likhitrakarn, Golovatch, Thach, Chhuoy, Ngor, Srisonchai, Sutcharit & Panha, 2020.

Among the Cambodian congeners, *Plusioglyphiulus battambangensis* sp. nov. and *P. parviserratus* sp. nov. represent clear geographic isolates, being remarkably disjunct from the more southernly distributed cave-dwelling species such as *P. boutini* Mauriès, 1970, *P. biserratus* Likhitrakarn et al., 2020, and *P. khmer* Likhitrakarn et al., 2020 (Fig. 12). Most importantly, the independent status of *P. battambangensis* sp. nov. is strongly supported by our molecular analysis; the uncorrected pairwise p-distance at the COI barcode locus yields a significant divergence of 10.3% from *P. parviserratus* sp. nov., a value fully corresponding to the stable species boundaries established for the family Cambalopsidae (Seesamut et al. 2026).

### Key to *Plusioglyphiulus* species currently known to occur in Cambodia

**Table:** 

1	All crests on collum undivided and mostly complete	***Plusioglyphiulus khmer* Likhitrakarn et al., 2020**
–	Crests on collum always divided and incomplete (Figs 3A, 3B, 4B, 6A–C, 6F, 6G, 8A, 8B, 9B, 11A–C, 11F, 11G)	**2**
2	General coloration very dark brown to blackish (fading to reddish after long conservation in alcohol). Paraprocts with a distinct, median, ridge-like elevation	***Plusioglyphiulus dubius* (Attems, 1938)**
–	General coloration lighter, usually yellow-brown to brown (Fig. 2A, B). Paraprocts flat medially	**3**
3	Male leg 1 telopodites 1-segmented	**4**
–	Male leg 1 telopodites 2-segmented (Figs 4D, 4E, 5A–D, 9D, 9E, 10A–D)	**5**
4	Male leg 1 with 1-segmented, very long telopodites, the latter almost as long as central hook. Anterior coxal process (ap) and paramedian coxal process (pp) of posterior gonopods suberect, long, slender, flagelliform, and clearly higher than Telopodite (te)	***Plusioglyphiulus biserratus* Likhitrakarn et al., 2020**
–	Male leg 1 telopodites 1-segmented, extremely short, nearly vestigial. Posterior gonopod telopodites otherwise shaped	***Plusioglyphiulus boutini* Mauriès, 1970**
5	Carinotaxy pattern of collum, (1a)/t+2p/t+3(3p/t)+4(4p/t)+ta/t+(5p)/t/t+ta/t+pp/(t)/t+m/m (Figs 3A, 3B, 4B, 6A–C, 6F, 6G); posterior gonopods with a stouter paramedian coxal process (pp) (Figs 4K, 5F, 5K–M) and a broad flagellum process (f) bearing a micro-serrate upper margin (Figs 4K, 4L, 5E–G, 5K–M)	***Plusioglyphiulus parviserratus* sp. nov**.
–	Carinotaxy pattern of collum, 1a/t+2p/t+3p/t/(t)+4p/(t)/t+(ta)/tc/t+5p/(t)/t+ta/t+pp/t/t+m/m (Figs 8A, 8B, 9B, 11A–C, 11F, 11G); posterior gonopods with a longer and more slender paramedian coxal process (pp) (Figs 9K, 10L, 10M) and a narrow flagellum process (f) bearing a smooth upper margin (Figs 9K, 9L, 10K–M)	***Plusioglyphiulus battambangensis* sp. nov**.

## Discussion

### Biogeography and species diversity of *Plusioglyphiulus* in Cambodia

According to the latest comprehensive catalogue of Cambodian Diplopoda (Likhitrakarn et al. 2015), also incorporating the two new species described herein, the millipede fauna of Cambodia remains strikingly depauperate, currently standing at a mere 28 recorded species distributed across 17 genera, 13 families, and 8 orders (Likhitrakarn et al. 2020a, 2025a; Srisonchai et al. 2020, 2023). With the addition of *Plusioglyphiulus parviserratus* sp. nov. and *P. battambangensis* sp. nov., the total number of *Plusioglyphiulus* congeners known from the country is raised to six (Fig. 12), while the global diversity of the genus expands to 32 valid species. It is worth emphasizing that all existing records of this genus are strictly confined to the southern and western karst regions of Cambodia.

Such a surprisingly low nominal diversity is undoubtedly rooted in an insufficient sampling effort that has failed to grasp the true richness of the local fauna, representing instead a profound collecting bias and a lack of systematic field explorations rather than actual ecological poverty. When contrasted with neighboring Thailand, where more than 14 *Plusioglyphiulus* species have been documented from more than 300 sampled localities (Likhitrakarn et al. 2023), the Cambodian karst systems appear vastly understudied. Given that Thailand harbors many more than a thousand surveyed caves, the discovery of only a handful of cave-dwelling millipedes in Cambodia underscores the urgent need for intensive speleobiological investigations across its isolated limestone hills.

### Karst isolation, micro-endemism, and transboundary faunal connections

The distribution patterns of the Cambodian *Plusioglyphiulus* heavily reinforce the concept of micro-endemism, a classic evolutionary trait observed in many cavernicolous and limestone-associated soil arthropods (Clements et al. 2006; Golovatch 2015). As illustrated in our updated distribution map (Fig. 12), the newly discovered taxa represent highly localized geographic isolates. The genus shows a profound propensity for parapatric or allopatric speciation driven by karst isolation; the severely fragmented nature of the limestone massifs in Battambang Province acts as an effective dispersal barrier, promoting rapid genetic divergence and morphogenetic specialization within individual cave systems or isolated mountain blocks. This geographic confinement and strict localization perfectly mirror the patterns observed in other highly specialized, karst-dwelling arthropod lineages across Indochina, where isolated limestone towers function as terrestrial islands that effectively halt gene flow (Clements et al. 2006; Srisonchai et al. 2018).

However, the syntopic or closely parapatric occurrence of related congeners within such localized environments warrants further eco-evolutionary consideration. Rather than assuming strict in-situ sympatric speciation, such distributional overlapping might heavily stem from complex, historical dispersal events from disparate microrefugia across shifting tropical corridors, culminating in subsequent secondary contact. While syntopy is a very rare phenomenon within the family Cambalopsidae, a notable parallel is documented in Borneo, where *Plusioglyphiulus bedosae* and *P. pallidior* stably coexist within the same cave system through marked body-size differentiation (Golovatch et al. 2009). A classic parallel in terms of montane habitat partitioning is observed in the high-montane forest fauna of northern Thailand, notably at Doi Inthanon and Doi Suthep, where up to ten sympatric species of the genus *Tylopus* Jeekel, 1968 successfully coexist, each maintaining a distinct phenological or microhabitat niche to avoid competitive exclusion (Likhitrakarn et al. 2010, 2014, 2016, 2025b). A similar ecological partition has also been well-documented in other Oriental paradoxosomatid lineages, such as *Touranella* Attems, 1937, where closely parapatric or sympatric congeners exhibit marked body-size differentiation and seasonal shifts in activity to mitigate intense ecological competition (Likhitrakarn et al. 2025c). We observed potential difference in body size between the two species, and niche differentiation remains a plausible explanation for their coexistence. However, this hypothesis requires ecological investigation with a larger data set. Further studies on microhabitat quantitative analysis are needed to better understand the mechanism underlying the coexistence of these two species.

In the case of the *Plusioglyphiulus* fauna treated herein, both new species demonstrate close morphological affinities with *P. likhitrakarni*, a species originally described from eastern Thailand (Golovatch et al. 2011). This morphological proximity strongly implies a historical transboundary faunal connection between the limestone karsts of eastern Thailand (e.g., Sa Kaeo and Chanthaburi provinces) and those of western Cambodia (Battambang Province). Such geographic patterns suggest that these regional karst systems might have shared historical ecological continuities prior to their more recent fragmentations.

Yet, despite their shared transboundary history and restricted distributions within the southern and western Cambodian karst systems, these micro-endemic species show profound, unequivocal morphological divergence. Most notably, they are easily distinguished from their syntopic or parapatric congeners, such as *P. boutini* Mauriès, 1970 and *P. khmer* Likhitrakarn, Golovatch, Thach, Chhuoy, Ngor, Srisonchai, Sutcharit & Panha, 2020, by the highly specialized architecture of the male gonopods and the specific carinotaxic ornamentation of the collum, providing explicit diagnostic features unique to each respective taxon.

### Imaging modalities in cambalopsid taxonomy

Modern millipede taxonomy increasingly relies on a combination of different imaging techniques to document and illustrate diagnostic somatic and gonopodal structures (Likhitrakarn et al. 2011, 2020b, 2022, 2024, 2025d, 2026a; Srisonchai et al. 2018; Huynh et al. 2023; Nguyen et al. 2023; Ng et al. 2025). In the present study, we evaluate and compare the utility, advantages, and limitations of four major modalities, including standard optical photography, line drawings, scanning electron microscopy (SEM), and micro-computed tomography (µCT) scanning, by utilizing the same cambalopsid species as a model.

Digital stereomicroscopic photography (Figs 3, 8) remains indispensable for capturing true-to-life coloration patterns and providing a comprehensive overview of the somatic habitus. However, even when deploying state-of-the-art optical systems, conventional light microscopy often fails to adequately resolve exceptionally minute, densely crowded, or overlapping appendages. Such limitations become particularly apparent when discerning the fine chaetotaxy on male leg-pairs 1–3, the intricate structures of the gnathochilarium, or the highly condensed, microscopic lamellae and processes characteristic of the anterior and posterior gonopods. Furthermore, subtle nuances of the cuticular sculpture, including the exact, detailed configuration of the metatergal ridges and crests, remain largely elusive under ordinary optical illumination.

Traditional line drawings, prepared with the aid of a camera lucida or executed from focus-stacked digital renderings, afford the ability to selectively emphasize and clarify specific diagnostic features. Nevertheless, the accuracy of such illustrations is heavily dependent upon the investigator’s personal interpretation and understanding of the structures under examination. This becomes particularly problematic when dealing with membranous or semi-translucent structures, which frequently remain visually elusive even under phase-contrast microscopy. For instance, the exact morphology of the bacilliform sensilla on antennomeres 5 and 6 (Figs 4C, 9C), the delicate penes on the male coxae 2 (Figs 4F, 9F), and especially the transparent flagellum process (f) (Figs 4K, 4L, 9K, 9L) on the posterior gonopods cannot be definitively resolved by means of standard light optics.

In sharp contrast, SEM provides unparalleled resolution and depth of field, bypassing any optical limitations associated with tissue translucency (Figs 5L, 5M, 10K–M). Because SEM utilizes an electron beam rather than light waves, the entire surface of the specimen is rendered with uniform, crisp focus, making it the gold standard for illustrating micro-characters. Its primary drawbacks, however, are that it provides no information regarding internal tissue density or variation in translucency within a single structure. Moreover, the prerequisite preparatory procedures (viz. critical point drying and gold sputter-coating) strictly demand structurally rigid specimens capable of withstanding high-vacuum conditions; otherwise, fragile or poorly calcified elements invariably risk distortion, fracturing, or artifactual displacement during the imaging process (e.g., Likhitrakarn et al. 2026b: fig. 13). The substantial maintenance costs and restricted availability of SEM facilities also present an obstacle to widespread, routine access.

Recently, micro-computed tomography (µCT scanning) has emerged as a powerful, increasingly accessible, and non-destructive alternative in myriapodology. In terms of external somatic architecture, the newly acquired 3DµCT data and their subsequent volume renderings yield highly satisfactory results (Figs 6, 11). The dataset provides a valuable non-destructive perspective, offering exceptional rotational capabilities and allowing for detailed observations of the cuticular patterns, rows of tubercles, and metatergal crests with remarkable clarity. Furthermore, virtual segmentation allows for the color-coded isolation of internal and closely appressed structures *in situ*, completely removing the necessity for physical dissection that might otherwise damage a unique specimen (Fig. 7A–D).

Nevertheless, regarding the visualization of intricate internal or highly localized structures, specifically the male leg-pairs 1–3 and the gonopods, the current resolution of non-invasive µCT data remains insufficient for critical taxonomic evaluation (Fig. 7E–M). The primary constraint inheres in the post-scanning segmentation and data processing, where manually delineating the boundaries and layers of densely packed structures is severely hampered by a lack of contrast and distinct separation. For instance, subtle micro-characters, such as the exact setation pattern on the mesal surface of leg 1 or the precise configuration of a vestigial second telopodite reduced to a mere rudimentary knob, cannot be successfully discriminated (Fig. 7E–H). Similarly, while the gonopod complex can be isolated as a distinct, color-coded volumetric mass (Fig. 7K–M), it fails to reveal the fine diagnostic features required for species-level discrimination and comparison, falling far short of the resolution captured via SEM (Fig. 5E–G). This limitation is undoubtedly exacerbated by the fact that the anterior and posterior gonopods remain naturally tightly appressed *in situ* (Figs 5E–G, 7A–D, 7K–M); without physical dissection prior to scanning, these overlapping elements mutually obscure one another, making the reconstruction of individual hidden surfaces from the virtual datasets exceedingly difficult.

In the present study, our post-processing of raw tomographic data benefited substantially from the advanced software suite AIVIA (v. 15) by Leica. While high-end commercial platforms like AIVIA yield vastly superior volumetric renderings and surface displays compared to standard open-source freeware, the inherent hardware and algorithmic thresholds required for resolving microscopic, naturally contiguous soft tissues or tightly appressed cuticular matrices remain a significant challenge.

Despite these spatial resolution constraints concerning the male genitalia, µCT scanning demonstrates immense potential for exploring structures deeply embedded within the body cavity, such as the female reproductive organ (vulva), which is situated internally within the third body segment. Given that male gonopods display hyper-diverse configurations routinely utilized to discriminate congeners, the degree of morphological variation in the corresponding female vulvae remains a fascinating, hitherto unresolved enigma in *Plusioglyphiulus* and related cambalopsids. Future refinements in µCT technology, scan geometries, and differential contrast-staining techniques will ultimately provide the keys to deciphering these internal structures.

To address the research objective regarding methodological efficiency, we present a qualitative comparison of the imaging methods applied in this study (Table 2). For standard identification, traditional light photography and line drawing continue to be the most economical but are limited by depth of field and subjective manual interpretation, respectively. SEM and µCT, in contrast, provide better non-destructive 3D visualization and high-resolution detail, but require much higher investments in equipment, specialized software and processing time. We conclude that a combined workflow that emphasizes routine photography for quick initial diagnosis, while leaving more advanced methods such as µCT and SEM for complex gonopod analysis, provides the best compromise between cost, time, and morphological resolution in cambalopsid taxonomy. As summarized in Table 2, this tiered approach allows researchers to select the imaging modality that best fits the specific taxonomic requirements of the specimen while effectively managing available resources and time constraints.

**Table 2.** Qualitative comparison of four imaging modalities used in the present study, highlighting methodological efficiency and morphological resolution.

**Imaging technique**	**Cost**	**Time consumption**	**Resolution / detail**	**Main limitation**
Light photography	Low	Low	Low/Moderate	Shallow depth of field
Line drawings	Low	Very High	High	Subjective interpretation
SEM	Moderate/High	Moderate	Very High	Destructive preparation
µCT	High	High	High/Very High	Processing complexity

### Conservation implications for cavernicolous ecosystems

Limestone karsts and their subterranean networks in Southeast Asia represent critical hotspots of global biodiversity, supporting exceptionally high numbers of narrow-range endemics. However, these specialized ecosystems are extremely fragile and increasingly threatened by commercial limestone mining for cement manufacture, agricultural development, and unmanaged cave tourism (Clements et al. 2006). Such anthropogenic activities greatly destabilize underground microclimates and deplete essential organic resources, especially bat guano, a fundamental substrate with which many cambalopsid millipedes are intimately connected (Golovatch 2015). Recent ecological studies of the caves of Cambodia have highlighted the significant conservation conflict that occurs due to human visitation and disturbance of the reproduction of insectivorous bats, where even small changes to the microenvironment can lead to immediate localized extinctions (e.g., Lim et al. 2018).

Being microendemics strictly confined to isolated karst towers, *Plusioglyphiulus parviserratus* sp. nov. and *Plusioglyphiulus battambangensis* sp. nov. are highly vulnerable to habitat degradation. Any structural compromise to their type localities could result in extinction before their ecological roles are fully understood, which is a vulnerability shared by many other cave-adapted arthropod lineages across Indochina. Therefore, basic taxonomic inventories are an essential first step toward assessing the biological value of individual karsts, providing the empirical baseline needed to advocate for sustainable cave management and formal site protection in Cambodia.

Regarding regional Cambalopsidae, we have merely touched the tip of the diversity iceberg (Golovatch et al. 2007b). Cambalopsids are exceptionally diverse across Southeast Asian karsts, showing strong ecological coupling with bat guano. Future systematic explorations, particularly within Cambodia’s under-sampled limestone formations, will undoubtedly reveal many more undescribed diplopods.

## Supplementary Material

XML Treatment for Plusioglyphiulus dubius

XML Treatment for Plusioglyphiulus boutini

XML Treatment for Plusioglyphiulus biserratus

XML Treatment for Plusioglyphiulus khmer

XML Treatment for Plusioglyphiulus parviserratus

XML Treatment for Plusioglyphiulus battambangensis

## Additional information

### Artificial Intelligence (AI) use

During the preparation of this manuscript, the authors used AI tools solely for English grammar correction and language polishing. No AI tools were used for data analysis, interpretation, or the generation of scientific conclusions. All research activities were conducted by the authors, who take full responsibility for the final content of the manuscript.

### Author ORCIDs

N. Likhitrakarn https://orcid.org/0000-0002-1306-317X

S.I. Golovatch https://orcid.org/0000-0001-7159-5484

R. Srisonchai https://orcid.org/0000-0002-7142-0999

S. Thi https://orcid.org/0009-0009-4827-1719

S. Chhin https://orcid.org/0000-0002-5612-7532

V. Lou https://orcid.org/0009-0004-8771-0378

P. Sinovas https://orcid.org/0000-0001-5396-8910

P. Jirapatrasilp https://orcid.org/0000-0002-5591-6724

C. Sutcharit https://orcid.org/0000-0001-7670-9540

S. Panha https://orcid.org/0000-0002-4431-2458

T. Seesamut https://orcid.org/0009-0003-9589-8641

### Data availability

All of the data that support the findings of this study are available in the main text.
